# Comparative Genomics for the Elucidation of Multidrug Resistance in Candida lusitaniae

**DOI:** 10.1128/mBio.02512-19

**Published:** 2019-12-24

**Authors:** Abhilash Kannan, Sandra A. Asner, Emilie Trachsel, Steve Kelly, Josie Parker, Dominique Sanglard

**Affiliations:** a Institute of Microbiology, Department of Laboratory, Lausanne University Hospital, Lausanne, Switzerland; b Unit of Pediatric Infectious Diseases and Vaccinology, Department of Paediatrics, Lausanne University Hospital, Lausanne, Switzerland; c Infectious Diseases Service, Department of Internal Medicine, Lausanne University Hospital, Lausanne, Switzerland; d Institute of Life Science, Swansea University Medical School, Swansea, United Kingdom; Duke University

**Keywords:** *Candida*, genome analysis, antifungal resistance, multidrug resistance

## Abstract

Multidrug resistance (MDR) has emerged in hospitals due to the use of several agents administered in combination or sequentially to the same individual. We reported earlier MDR in Candida lusitaniae during therapy with amphotericin B (AmB), azoles, and candins. Here, we used comparative genomic approaches between the initial susceptible isolate and 4 other isolates with different MDR profiles. From a total of 18 nonsynonymous single nucleotide polymorphisms (NSS) in genome comparisons with the initial isolate, six could be associated with MDR. One of the single nucleotide polymorphisms (SNPs) occurred in a putative transcriptional activator (*MRR1*) resulting in a V668G substitution in isolates resistant to azoles and 5-fluorocytosine (5-FC). We demonstrated by genome editing that *MRR1* acted by upregulation of *MFS7* (a multidrug transporter) in the presence of the V668G substitution. *MFS7* itself mediated not only azole resistance but also 5-FC resistance, which represents a novel resistance mechanism for this drug class. Three other distinct NSS occurred in *FKS1* (a glucan synthase gene that is targeted by candins) in three candin-resistant isolates. Last, two other NSS in *ERG3* and *ERG4* (ergosterol biosynthesis) resulting in nonsense mutations were revealed in AmB-resistant isolates, one of which accumulated the two *ERG* NSS. AmB-resistant isolates lacked ergosterol and exhibited sterol profiles, consistent with *ERG3* and *ERG4* defects. In conclusion, this genome analysis combined with genetics and metabolomics helped decipher the resistance profiles identified in this clinical case. MDR isolates accumulated six different mutations conferring resistance to all antifungal agents used in medicine. This case study illustrates the capacity of C. lusitaniae to rapidly adapt under drug pressure within the host.

## Abstract (precis)

Antifungal resistance is an inevitable phenomenon when fungal pathogens are exposed to antifungal drugs. These drugs can be grouped in four distinct classes (azoles, candins, polyenes, and pyrimidine analogs) and are used in different clinical settings. Failures in therapy implicate the sequential or combined use of these different drug classes, which can result in some cases in the development of multidrug resistance (MDR). MDR is particularly challenging in the clinic since it drastically reduces possible treatment alternatives. In this study, we report the rapid development of MDR in Candida lusitaniae in a patient, which became resistant to all known antifungal agents used until now in medicine. To understand how MDR developed in C. lusitaniae, whole-genome sequencing followed by comparative genome analysis was undertaken in sequential MDR isolates. This helped to detect all specific mutations linked to drug resistance and explained the different MDR patterns exhibited by the clinical isolates.

## INTRODUCTION

Candida (teleomorph Clavispora) lusitaniae is a ubiquitous ascomycetous yeast that can be recovered from soils and water but can survive in different hosts (birds and mammals). It is known as an opportunistic haploid yeast and is the cause of infrequent candidemia (1). Mortality due to C. lusitaniae fungemia varies extensively (5% to 50%) and has often been associated with resistance to amphotericin B (AmB) (2). Even if C. lusitaniae is considered susceptible to most systemic antifungal agents, several reports have documented the development of antifungal resistance. Usually, antifungal resistance is restricted to a single agent. For example, Desnos-Ollivier et al. (3) reported candin resistance in C. lusitaniae from a patient treated with caspofungin over 11 to 17 days. Resistance was associated with a missense mutation (S645F) in the *FKS1* gene encoding 1,3-β-glucan synthase, the target of candins in several fungal species. 5-Fluorocytosine (5-FC) resistance has also been described in C. lusitaniae and is associated with defects in cytosine permease (4). Recently, C. lusitaniae resistance to fluconazole (FLC) was reported in the lungs of cystic fibrosis patients, even in the absence of a specific treatment with this drug (5). Simultaneous resistance to AmB and FLC was described in a patient treated intermittently with the two agents (6). We reported that C. lusitaniae can develop multidrug resistance (MDR), which is understood as resistance to at least 2 different drug classes. MDR occurred in 4 distinct C. lusitaniae isolates recovered from a patient treated sequentially with different antifungal drugs, including azoles, AmB, and candins (7). Antifungal pressure selected distinct MDR profiles that were corresponding to the administered antifungals. The systemic sequential isolates that were obtained from the treated patient were shown to be related to each other, suggesting that they had a common ancestor. We undertook earlier the identification of mutations associated with these drug resistance patterns (7), and only three separate mutations in *FKS1* resulting in candin resistance were identified. Here, we applied whole-genome sequencing approaches in sequential isolates of the treated patient and compared them with the most susceptible isolate that was recovered at early stages of therapy. Genome comparisons revealed several genome alterations explaining the different antifungal resistance profiles of the clinical strains and genetic approaches confirmed their roles in antifungal resistance.

## RESULTS

### C. lusitaniae genomes.

**FIG 1:**
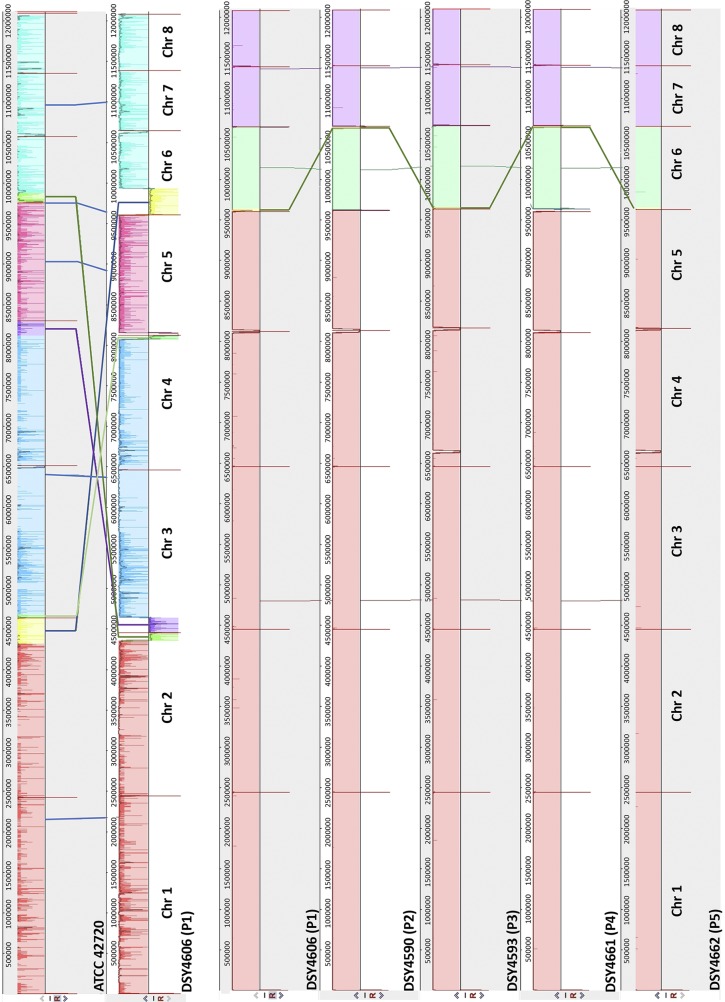
Whole-genome alignments. At the top is shown the alignment between ATCC 42720 and isolate P1, and the rows below that show the alignment between isolates P1 to P5. Alignments were obtained using Mauve (version 2015-02-25), with default parameters, and the aligner software Muscle 3.6. Vertical red bars indicate separations between the 8 chromosomes. Colored crossed lines between chromosomes of individual isolates indicate major translocations. Isolate designations are given both as the laboratory collection name and as earlier published (7) (in parentheses). Color intensities between chromosomes indicate SNP densities between comparisons.

We determined earlier that the C. lusitaniae sequential isolates (named P1 to P5) recovered from the treated patient were related to each other using restriction sites polymorphism approaches. Isolate P1 was the earliest but still drug-susceptible isolate, while the others (P2 to P5) exhibited MDR (7). To enable complete comparisons between these strains, we carried out their genome sequencing using PacBio technology to produce large telomere-to-telomere assemblies. Five independent assemblies were obtained with the five isolates containing each eight major contigs (smaller contigs corresponding to mitochondrial genomes were ignored). These 8 major contigs were likely to correspond to the eight suggested chromosomes of C. lusitaniae previously described either by supercontig assemblies of C. lusitaniae ATCC 42720 (8) or by chromosome separation from electrophoresis (9) and centromere mapping (10). These PacBio contigs were designated Chr 1 to Chr 8 and were sorted by their sizes (see Table S2 in the supplemental material). We first compared the genomes of strain P1 with ATCC 42720 by whole-genome alignments. Several chromosome rearrangements were observed between the two genomes (Fig. 1A) but principally occurring at chromosome ends between Chr 2 and Chr 6 and between Chr 3 and Chr 4. Genome alignments between isolates P1 to P5 did not reveal major rearrangements, except for a terminal chromosome fragment of Chr 6, which was positioned at the opposite chromosome ends in isolates P2 and P4 (Fig. 1B). In addition, P2 and P5 isolates exhibited a sequence gap at the left side of Chr 4 (Fig. 1B), which was the result of a 30-kb fragment duplication in isolates P1, P3, and P4 (see below).

10.1128/mBio.02512-19.4 TABLE S2 Chromosome lengths of C. lusitaniae chromosomes. Download Table S2, DOCX file, 0.1 MB. Copyright © 2019 Kannan et al. 2019 Kannan et al. This content is distributed under the terms of the Creative Commons Attribution 4.0 International license.

10.1128/mBio.02512-19.5 FILE S1 SNP counts between ATCC 42720 and isolate P1, Chr 1 to Chr 3. Download File S1, XLSX file, 2.1 MB. Copyright © 2019 Kannan et al. 2019 Kannan et al. This content is distributed under the terms of the Creative Commons Attribution 4.0 International license.

10.1128/mBio.02512-19.6 FILE S2 SNP counts between ATCC 42720 and isolate P1, Chr 4 to Chr 8. Download File S2, XLSX file, 2.7 MB. Copyright © 2019 Kannan et al. 2019 Kannan et al. This content is distributed under the terms of the Creative Commons Attribution 4.0 International license.

10.1128/mBio.02512-19.7 FILE S3 Transposons and LTRs of C. lusitaniae and *GRP2* homologs in C. lusitaniae. Download File S3, XLSX file, 0.1 MB. Copyright © 2019 Kannan et al. 2019 Kannan et al. This content is distributed under the terms of the Creative Commons Attribution 4.0 International license.

10.1128/mBio.02512-19.8 FILE S4 Nucleotide differences between isolates P1 to P5. Download File S4, XLSX file, 1.2 MB. Copyright © 2019 Kannan et al. 2019 Kannan et al. This content is distributed under the terms of the Creative Commons Attribution 4.0 International license.

10.1128/mBio.02512-19.9 FILE S5 RNA-seq data comparisons between isolates P3 and P4 versus isolate P1. Download File S5, XLSX file, 0.1 MB. Copyright © 2019 Kannan et al. 2019 Kannan et al. This content is distributed under the terms of the Creative Commons Attribution 4.0 International license.

10.1128/mBio.02512-19.10 FILE S6 Antifungal susceptibility data. Download File S6, XLSX file, 0.1 MB. Copyright © 2019 Kannan et al. 2019 Kannan et al. This content is distributed under the terms of the Creative Commons Attribution 4.0 International license.

To allow further genome comparisons, genome data of isolate P1 were next subjected to gene annotations (see Materials and Methods). This annotation served as a basis for P2 to P5 genome annotations. The genome characteristics of P1 to P5 are supplied in Table 1. The number of detected coding sequences (CDS) were ranging from 5,662 to 5,684 in the P1 to P5 genomes. These numbers lie within ranges estimated in genomes of ATCC 42720 and CBS 6936 (Table 1). The genome of CBS 6936 was recently reported but did not reach the level of whole-chromosome assemblies (11). Given that the P1 and ATCC 42720 genomes were chromosome assemblies, these two genomes were systematically compared for the occurrence of single nucleotide polymorphisms (SNPs) resulting in synonymous and nonsynonymous changes in the encoded proteins (Files S1 and S2). Our data suggested the existence of 83,225 SNPs between the CDS of these two genomes. The count of non-CDS SNPs was up to 118,174 between the two strains, which results in an SNP density of about 1 SNP per 60 bp. This density is higher than that observed between Candida isolates of the same species (8). A density plot of the detected SNPs along the chromosomes revealed regions of high SNP densities separated by stretches of low densities which are indicative of recombination (Fig. S1). C. lusitaniae is one of the few *Candida* spp. that is able to undergo mating and meiosis; therefore, it can be anticipated that recombination events could be traced to some extent (12). Similar conclusions deduced from SNP density plots were drawn by genome comparisons of C. glabrata genomes (13). P1 is of the alpha mating type, which is prevalent in this species (14); thus, mating may have occurred at some point during the evolution of this strain.

**TABLE 1:** Genome characteristics of C. lusitaniae isolates

Isolate^*a*^	No. of:				Reference or source
	CDS	Genes	tRNA	rRNA	
DSY4606 (P1)	5,676	5,882	197	9	This study
DSY4590 (P2)	5,662	5,869	196	11	This study
DSY4593 (P3)	5,684	5,892	198	10	This study
DSY4661 (P4)	5,679	5,886	197	10	This study
DSY4662 (P5)	5,676	5,883	197	10	This study
ATCC 42720	5,936	6,154	217	NA^*b*^	8
CBS 6936	5,539	5,740	197	4	11

a Isolate designations are given both as the laboratory collection name and as published previously in parentheses (7).

b NA, not available.

10.1128/mBio.02512-19.1 FIG S1 SNP density plots from comparisons between C. lusitaniae isolate P1 and ATCC 42720. Red bars correspond to the lengths of each indicated chromosome. Download FIG S1, PDF file, 0.7 MB. Copyright © 2019 Kannan et al. 2019 Kannan et al. This content is distributed under the terms of the Creative Commons Attribution 4.0 International license.

A few other characteristics were observed in the genomes of P1 to P5. As mentioned above, a 30-kb region in the Chr 4 in P1, P3, and P4 was duplicated compared to P2 and P5. This region contains 13 CDS and among them a putative agglutinin of 2,633 amino acids similar to Als2 from Candida albicans. The significance of this duplication remains unclear. In addition, the P1 to P5 isolates each contain 15 separate retrotransposon-like elements divided into 4 different groups, some of which are flanked by nucleotide repeats referred to as long terminal repeats (LTRs) (File S3). So far, the presence of retrotransposons has not been firmly reported in this species. Last, we observed the expansion of specific protein types in each of these isolates. For example, seven distinct proteins similar to the NADPH-dependent methylglyoxal reductase *GRP2* from C. albicans were detected (File S3). *GRP2* in C. albicans was shown to be involved in oxidative stress response (15) and is coregulated with other azole-responsive genes (16). The C. lusitaniae *GRP2*-like genes in P1 to P5 exhibit 94 to 58% similarity with each other. So far, no equivalent of gene family expansion in this type of genes was reported in other *Candida* species.

### Comparative analysis between the P1 to P5 genomes.

In order to identify mutations associated with drug resistance in the sequenced C. lusitaniae genomes, all five genomes were aligned, and nucleotide differences in coding and noncoding regions were recorded, taking isolate P1 as a reference (see Materials and Methods). We showed earlier that isolate P1 was likely to be the parent of all subsequently isolated C. lusitaniae samples from the treated patient (isolates P2 to P5). Table 2 summarizes the occurrence of nucleotide changes existing between isolates P2 to P5, taking P1 as a genome reference. In general, one can observe a low level of variation between the genomes, even if yeast samples were recovered within a 5-month period. A total of 18 nonsynonymous SNPs were observed, among which 13 were unique. We counted 166 SNPs/indels in intergenic sequences between the isolates and 14 other indels in open reading frames (ORFs), some of them (five) causing unique frameshifts and CDS truncations (File S4).

**TABLE 2:** Occurrence of nucleotide changes in isolates P2 to P5 compared to P1

Contig or total	No. of changes by isolate for:											
	Nonsynonymous SNPs (*n* = 18)				Intergenic SNPs/indels (*n* = 166)				ORF indels/frameshifts (*n* = 14)			
	P2	P3	P4	P5	P2	P3	P4	P5	P2	P3	P4	P5
Chr 1	1	2	2	1	9	6	6	6	1	0	1	1
Chr 2	2	0	1	2	9	5	6	3	0	1	2	1
Chr 3	0	3	0	0	10	5	5	6	0	1	0	0
Chr 4	0	0	0	0	15	11	8	9	0	1	2	0
Chr 5	0	1	0	0	5	4	4	3	0	1	0	0
Chr 6	0	0	0	3	2	2	2	1	0	0	1	1
Chr 7	0	0	0	0	3	3	2	2	0	0	0	0
Chr 8	0	0	0	0	6	4	2	2	0	0	0	0
Total	3	6	3	6	59	40	35	32	1	4	6	3

Table 3 includes the 25 different ORFs of isolates P2 to P5 that differ from isolate P1. Two in-frame insertions occurred in two proteins similar to transcription factors (*CZF1* and *SPT4*) in isolates P3 and P5. These insertions could potentially exert positive or negative effects on the functions of these factors, which remains to be addressed. Other insertions occurred in isolates P2, P4, and P5 in proteins similar to those encoded by *BPH1* (a protein playing a role in protein sorting), *BNR1* (cytoskeleton protein), *CBP1* (corticosteroid-binding protein), and to a protein with unknown function (similar to CLUG_03676 from C. lusitaniae ATCC 42720). Six frameshift mutations occurred in these genome comparisons. Interestingly, two of them were proteins with potential functions in cell wall biogenesis. The first one was a frameshift mutation in EJF17_2079 (isolate P4) with similarity to *UTR2* (extracellular glycosidase), resulting in a truncation of the 26 amino acids of the full protein. The significance of this alteration is unknown; however, the C-terminal end of Utr2p contains a signal sequence important for glycosylphosphatidylinositol (GPI) anchoring of the protein. Therefore, one can speculate some functional consequence of this protein truncation in isolate P4. The second one was a frameshift mutation in EJF15_20956 and EJF17_20956 from isolates P3 and P4 with similarity to *GAS4* (a 1,3-beta-glucanosyltransferase). This mutation also results in a truncation of the full protein at the C terminus but of only three amino acids. Gas4p contains also a C-terminal signal sequence important for GPI anchoring of the protein. This short truncation may have limited impact on protein function; however, this needs still further verification. The four other frameshift mutations occurred in ORFs with other functions in P2 to P5 isolates, all resulting in protein truncations. It is difficult to predict to which extent these truncations could affect the current phenotypic characterization of the recovered isolates.

**TABLE 3:** ORF alterations in isolates P1 to P5 compared to P1

P2 to P4 altered locus/loci	Isolate(s)	Gene similarity^*a*^	Description	Amino acid change^*b*^	Protein effect	CDS codon no.^*c*^
EJF18_20133	P2, P5	ERG4_YEAST	Delta[24(24(1))]-sterol reductase	S412→*	Truncation	412
EJF18_60340	P5	ERG3_CANAX	Delta(7)-sterol-5(6)-desaturase	Q308→*	Truncation	308
EJF15_10551, EJF17_10551	P3, P4	MRR1_CANAL	Multidrug resistance regulator 1	V→G	Substitution	668
EJF15_10714, EJF17_10714, EJF18_10714	P3, P4, P5, P2	BSC5_YEAST	Bypass of stop codon protein 5	F→L	Substitution	447
EJF17_20482	P2	FKS1_YEAST	1,3-β-glucan synthase component FKS1	S→Y	Substitution	638
EJF16_20482	P4	FKS1_YEAST	1,3-β-glucan synthase component FKS1	S→Y	Substitution	631
EJF18_20482	P5	FKS1_YEAST	1,3-β-glucan synthase component FKS1	S→P	Substitution	638
EJF15_30508	P3	OPT2_YEAST	Oligopeptide transporter 2	P→L	Substitution	895
EJF15_30569	P3	MSH2_YEAST	DNA mismatch repair MSH2	V→G	Substitution	186
EJF15_30782	P3	CLUG_02496	60S ribosomal protein	E→K	Substitution	206
EJF15_50669	P3	CLUG_04164	CLUG_04164 Clavispora lusitaniae ATCC 42720	P→S	Substitution	25
EJF18_60098	P5	CBP1_CANAL	Corticosteroid-binding protein	A→K	Substitution	531
EJF18_60098	P5	CBP1_CANAL	Corticosteroid-binding protein	K→A	Substitution	536
EJF18_20361	P5	STP4_CANAL	Transcriptional regulator STP4	QF→QFQYPGGTKKSQRNQVAGCCRLCQAQF	Insertion	306
EJF15_30283	P3	CZF1_CANAL	Zinc cluster transcription factor CZF1	N→NN	Insertion	150
EJF17_40359	P4	BPH1_YEAST	Beige protein 1	EE→E	Deletion	1181
EJF17_40476	P4	CLUG_03676	CLUG_03676 Clavispora lusitaniae ATCC 42720	Q→QQ	Insertion	141
EJF16_40795	P2	BNR1_CANAL	Formin BNR1	HEAKD→H	Deletion	1777
EJF18_60098	P5	CBP1_CANAL	Corticosteroid-binding protein	D→DKEDRD	Insertion	540
EJF17_10341	P4	YD338C_YEAST	Uncharacterized transporter YDR338C	S39	Frameshift	39
EJF18_10886, EJF16_10886	P5, P2	CLUG_00859	CLUG_00859 Clavispora lusitaniae ATCC 42720	G89	Frameshift	89
EJF17_20798	P4	UTR2_CANAL	Extracellular glycosidase UTR2	V401	Frameshift	401
EJF15_20956, EJF17_20956	P3, P4	GAS4_YEAST	1,3-β-glucanosyltransferase GAS4	P175	Frameshift	175
EJF15_50735	P3	LGUL_YEAST	Lactoylglutathione lyase	Y268	Frameshift	268
EJF17_60452	P4	APE2_CANAL	Aminopeptidase 2	L725	Frameshift	725

a Similarities to proteins of the UniProt database are given. The UniProt database contains protein names listing the encoding gene names followed by suffixes indicating the species origin (CANAL and CANAX, Candida albicans; YEAST, Saccharomyces cerevisiae). Some matches with C. lusitaniae ATCC 42720 are given with the original nomenclature.

b *, conversion of the codon into a stop codon. For single amino acids, the position of the last amino acid of the coding sequence after insertion/deletion of nucleotides is indicated.

c CDS positions at which the amino acid changes occur.

With regard to amino acid substitutions detected in the genome comparisons, we first observed changes in four ORFs, including EJF15_30508 (similar to *OPT2*, an oligopeptide transporter), EJF15_30569 (similar to *MSH2*, a DNA mismatch repair gene), EJF15_30782 (similar to a 60S ribosomal protein), EJF15_50669 (similar to the hypothetical protein CLUG_04164 from C. lusitaniae ATCC 42720), and EJF18_60098 (similar to *CBP1*, a corticosteroid-binding protein gene). Out of these, the change V186G in EJF15_30569 (similar to *MSH2*) from isolate P3 if of potential interest. Yeast *MSH2* is part of a complex involved in DNA repair, and *MSH2* mutations were shown to contribute to increasing mutation rates in several fungal species (17, 18). The V186G substitution in EJF15_30569 lies near a domain referred to as the connector domain that is important for the function of Msh2p (19). It is therefore possible that the V186G substitution from isolate P3 affects Msh2p function and consequently the frequency at which mutations can occur in isolate P3 compared to P1.

While inspecting these data from the perspective of antifungal drug resistance, mutations in several genes associated with the specific drug resistance profiles of P2 to P5 isolates could be highlighted and are summarized below.

### (i) V668G in EJF15_10551 and EJF17_10551 from isolates P3 and P4.

EJF15_10551 and EJF17_10551 encode a protein similar to MRR1_CANAL (multidrug resistance regulator 1 of C. albicans). In C. albicans, this protein is known as a regulator of *MDR1*, a major facilitator efflux transporter that mediates FLC resistance (20). Gain-of-function (GOF) mutations in *MRR1* result in strong upregulation of this transporter and, consequently, azole resistance. *MFS7* (a homolog of *MDR1* in C. lusitaniae) was previously reported to be upregulated in isolates P3 and P4 compared to P1 (7). We therefore propose that the V668G substitution in EJF15_10551 and EJF17_10551 (now referred to as *MRR1*) is a GOF mutation that mediates *MFS7* upregulation in P3 and P4.

### (ii) S638Y, S631Y, and S638P in EJF17_20482, EJF16_20482, and EJF18_20482 from P2, P4, and P5.

EJF17_20482, EJF16_20482, and EJF18_20482 encode proteins similar to FKS1_YEAST (1,3-β-glucan synthase). Fks1p is a 1,3-β-glucan synthase, a critical enzyme in the biosynthesis of 1,3-β-glucans in fungi. It is also the target of candins (21). We reported earlier the presence of these distinct mutations in C. lusitaniae isolates P2, P4, and P5 and showed that they were causing candin resistance but using Saccharomyces cerevisiae as a surrogate system (7). The current sequencing data confirm these different mutations in EJF17_20482, EJF16_20482, and EJF18_20482 (now referred as to *FKS1*) that were previously deduced from specific Sanger sequencing reactions.

### (iii) Truncations in EJF18_20133 and EJF18_60340 from isolates P2 and P5.

EJF18_20133 and EJF18_60340 encode proteins similar to ERG4_YEAST [delta(24(24(1)))-sterol reductase] and ERG3_CANAX [delta(7)-sterol-5(6)-desaturase], respectively. In isolate P2, EJF18_20133 (further referred as to *ERG4*) underwent a nucleotide change (C to A) converting the TCG codon (Ser^412^) into a stop codon (TAG; *erg4^amber^*), thus truncating the full Erg4p by 49 amino acids. While the same mutation was detected in isolate P5, another distinct mutation in EJF18_60340 (now referred as to *ERG3*) was revealed in the same isolate. *ERG3* underwent a nucleotide change (C to T) converting the CAA codon (Gln^308^) into a stop codon (TAA; *erg3^ochre^*), thus truncating the full Erg3p by 59 amino acids. Both P2 and P5 were reported to be resistant to AmB (7). Both *ERG4* and *ERG3* truncations may have resulted in altered sterol composition in these isolates, thus leading to the depletion of ergosterol, which is a known factor mediating AmB resistance (22).

**FIG 2:**
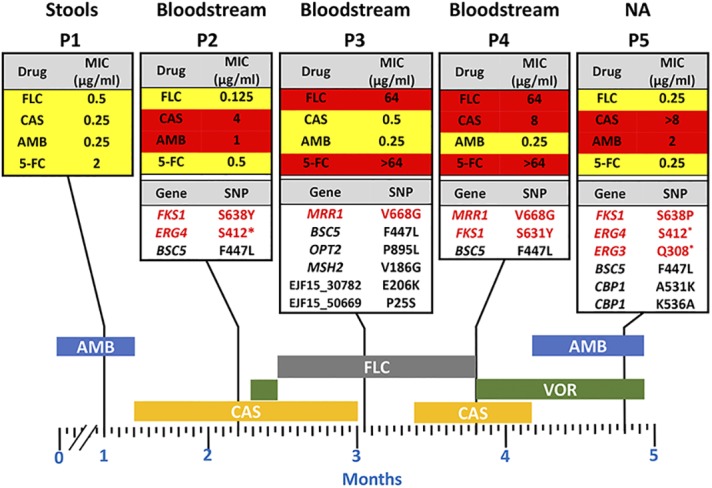
Overview of C. lusitaniae isolates phenotypes and genotypes. A timeline scale indicates the drug regimen administered to the patient, as reported earlier (7). Vertical black bars highlight the timing of sampling. Each fungal isolate (P1 to P5) is documented for drug susceptibilities and the occurrence of SNPs in CDS and origin of sampling. NA, not available; VOR, voriconazole; CAS, caspofungin; FLC, fluconazole, AMB, amphotericin B. Wild-type drug MICs are boxed in yellow, while MICs indicating resistance are boxed in red. Only SNP variants in coding regions are reported here. Red-labeled genes and their SNPs indicate associations to drug resistance. S412* and Q308* signify changes of serine and glutamine, respectively, into a stop codon.

Taking these comparative descriptions together, we summarized the potential associations existing between the isolate genotypes and the reported antifungal drug resistance profiles in Fig. 2, considering only single-amino-acid substitutions for comparisons. Isolate P1 was previously shown as the earliest yeast recovered from the patient at early stages of antifungal treatment, and isolates P2 to P5 were timely sequential isolates. Given the high nucleotide resemblance between the isolates and that isolates P2 to P5 exhibit the same SNP as P1 (*BSC5* F447L), the data confirm that P1 is the ancestor of P2 to P5. However, it is less likely that each isolate represents a progeny from the other. This conclusion is supported by the different SNP profiles found in the isolates (Table 3). Interestingly, P3 and P4 share the same substitution in *MRR1* (V668G) and the same frameshift mutation in EJF15_20956 and EJF17_20956 (*GAS4*), thus pointing to a common ancestor. P2 and P5 also share SNPs leading to the same truncation in *ERG4* (S412*) and the same frameshift mutation in EJF14_10886, which suggests that the two isolates originated from a common ancestor. However, each of these isolates also contains specific other SNPs, indicating that they evolved in individual directions. Fitness costs could have been a consequence of the different mutations occurring during the evolution of the clinical strains. It cannot be excluded that these possible fitness defects were compensated for by distinct mutations in isolates P2 to P5 listed in Table 3. To summarize, besides the already-known reported *FKS1* mutations, we identified here three novel mutations, V668G in *MRR1*, possibly mediating FLC resistance; S412* in *ERG4*; and Q308* in *ERG3*, which might be responsible for AmB resistance.

### Role of *MRR1* in FLC resistance.

Our data suggested that *MRR1* (EJF14_10551) could be involved in FLC resistance in C. lusitaniae due to the occurrence of a V668G substitution. To address this question, we first undertook an RNA sequencing (RNA-seq) analysis in isolates P3 and P4 grown under normal conditions and compared the data with P1 (see Materials and Methods). According to our selection (1.5-fold expression change compared to P1; File S4), there were 54 and 30 genes up- and downregulated, respectively, in P4. In a comparison of P3 with P1, 16 genes were inversely regulated, keeping this threshold. Using a Gene Ontology (GO) terms enrichment analysis based on gene annotations of C. albicans homologs, significant enrichment of functions related to azole transport and oxidoreductase activity were detected among commonly upregulated genes in P3 and P4 (File S4). Highly upregulated genes (215- to 57-fold in P4 and P3 versus P1) involved in these biological processes include alcohol dehydrogenases (EJF14_60130 and EJF14_40004, similar to *ADH6* and *ADH7*) and a NADPH-dependent methylglyoxal reductase (EJF14_20072, similar to *GRP2*). *MFS7* was among these highly upregulated genes (17- and 15-fold upregulation in P4 and P3 versus P1) in isolates containing the *MRR1* V668G substitution. *MRR1* itself was significantly upregulated in P3/P4 isolates (>1.5-fold versus P1). These expression profiles are consistent with the imprint of *MRR1* GOF mutations in C. albicans on the transcriptome of this species (20). We therefore suggest that the C. lusitaniae *MRR1* V668G substitution is a GOF mutation which impacts on the expression of different target genes, among them *MFS7*.

**FIG 3:**
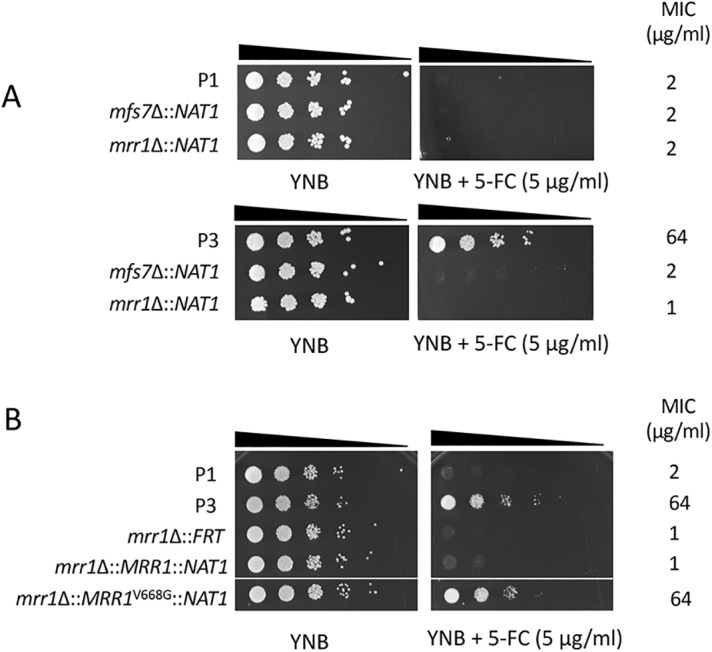
*MRR1* and *MFS7* mediate FLC resistance in C. lusitaniae. (A) Tenfold serial dilutions were performed, starting with an inoculum of about 10^5^ cells. Mutants for *MRR1* and *MFS7* in P3 correspond to DSY5240 and DSY5242 and in P1 to isolates DSY5246 and DSY5248, respectively. MIC values were obtained by MIC measurements using the SYO system, as described in Materials and Methods. (B) Reversion of *MRR1* deletion. Mutants for *MRR1* correspond to DSY5416. Revertants for *MRR1* wild-type allele and *MRR1* GOF allele (*MRR1*^V668G^) correspond to isolates DSY5437 and DSY5438, respectively (Table S1). The white line indicates removal of a yeast sample from the original agar plate.

**FIG 4:**
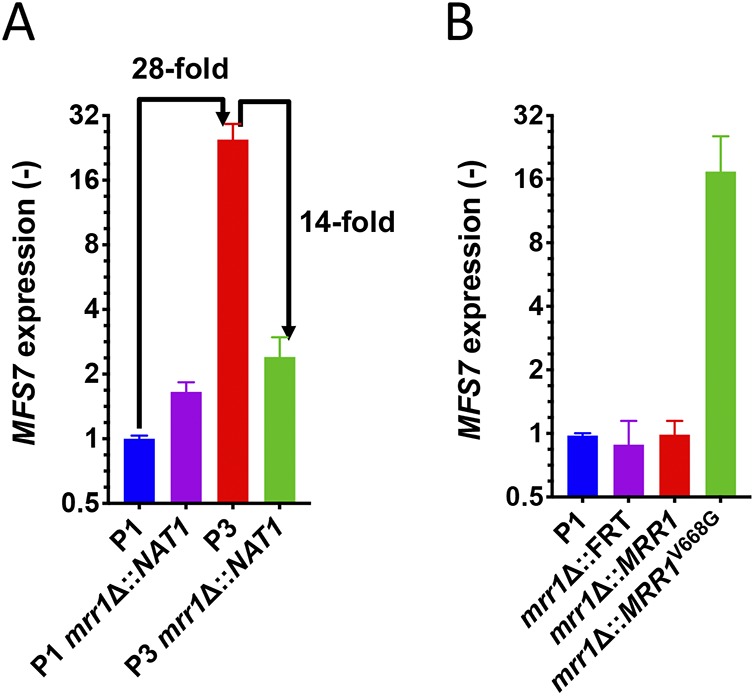
Expression of *MFS7* in C. lusitaniae. (A) *MFS7* expression in P1 and P3 and derived *MRR1* mutants (DSY5246 and DSY5240, respectively). *MFS7* expression fold changes are indicated between specific isolates. (B) *MFS7* expression in *MRR1* revertants (for DSY5437, *mrr1*Δ::*MRR1*, and for DSY5438, *mrr1*Δ::*MRR1*^V668G^) derived from DSY5416 (*mrr1*Δ::FRT) (Table S1). *MFS7* expression was calculated relative to the initial isolate P1. qPCRs were performed with biological triplicates, as described in Materials and Methods.

In order to further address the role of *MRR1* and *MFS7* in the drug resistance profiles, we first attempted gene knockout approaches. Preliminary results using homologous recombination with a dominant marker (*SAT1*) flanked by *MFS7* and *MRR1* 5′ and 3′ regions proved to be inefficient. We therefore used a CRISPR-Cas9 approach with guide RNAs specific for *MFS7* and *MRR1* using *in vitro*-reconstituted RNA-protein complexes (RNPs) and repair fragments with the dominant marker by *NAT1* flanked by *MFS7* and *MRR1* 5′ and 3′ regions (see Materials and Methods). CRISPR-Cas9 and corresponding repair fragments were used in isolates P1 and P3, and the expected mutants were verified as described (see Materials and Methods). Figure 3A shows that the deletion of both *MFS7* and *MRR1* in the background of azole-susceptible isolate P1 did not significantly alter FLC susceptibility assessed by serial dilution assays and MIC measurements (MIC values fluctuated between 1 and 0.25 μg/ml FLC for all P1 and P1-derived mutants; see File S6 for full MIC data). On the contrary, deletion of both *MFS7* and *MRR1* in the background of the azole-resistant isolate P3 significantly altered azole susceptibility, as follows: while the initial isolate P3 exhibited a MIC value of 64 μg/ml FLC, deletion of *MRR1* resulted in a 64-fold MIC decrease (FLC MIC, 1 μg/ml; File S6), and the deletion of *MFS7* resulted in a 8-fold MIC decrease (FLC MIC, 8 μg/ml; File S6). We addressed the effect of *MRR1* deletion on *MFS7* expression by quantitative PCR (qPCR) and, while *MFS7* expression was increased by 28-fold between the isolates P1 and P3, it was decreased by 14-fold in the absence of *MRR1* between P3 and its *MRR1*-derived mutant (Fig. 4A). These findings indicate that the *MRR1* allele of P3 containing the V668G substitution mediates *MFS7* regulation in C. lusitaniae.

10.1128/mBio.02512-19.3 TABLE S1 Primers, guides, and strains used in this study. Download Table S1, DOCX file, 0.1 MB. Copyright © 2019 Kannan et al. 2019 Kannan et al. This content is distributed under the terms of the Creative Commons Attribution 4.0 International license.

To demonstrate a genetic link between the presence of a *MRR1* GOF mutation and azole resistance, we next constructed revertants from a *MRR1* mutant in which the *MRR1* alleles from P1 (wild-type allele) and P3 (GOF allele) were reintroduced. This experiment required the recycling of the *NAT1* dominant marker; thus, a *MAL2*-dependent flipper system permitting excision of the *NAT1* marker was designed, and a separate *MRR1* mutant lacking the selection marker was produced (DSY5416, see Materials and Methods). CRISPR-Cas9 combining two guide RNAs targeting flanking *MRR1* regions was used with *MRR1* wild-type and GOF alleles on the *NAT1* dominant marker to produce the final revertants. Figure 3B shows that the presence of the *MRR1* GOF allele alone could restore azole resistance (FLC MIC, 64 μg/ml; File S6) and thus demonstrates the association between the *MRR1* GOF mutation V668G and azole resistance. Consistently, high *MFS7* expression was restored from the *MRR1* deletion mutant in the presence of the *MRR1* GOF mutation and not with the *MRR1* wild-type allele (Fig. 4B).

### Role of *MRR1* in 5-FC resistance.

**FIG 5:**
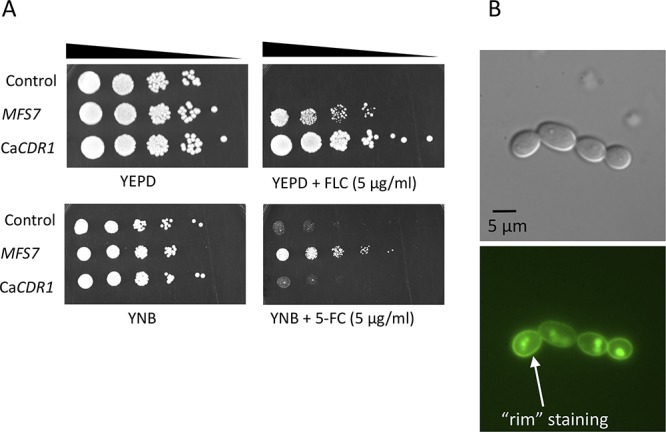
*MFS7* mediates 5-FC resistance. (A) Tenfold serial dilutions were performed, starting with an inoculum of about 10^5^ cells at the indicated 5-FC concentration. *MFS7* overexpression was obtained by a *CDR1*-MFS7 chimeric construct expressed in DSY5170. The control strain is DSY5169 (see Table S1), and the *CDR1* overexpression strain is ANY-MDR1-GFP (Ca*CDR1*) (Table S1). (B) Localization of MFS7-GFP in C. albicans. Microscopy was performed under normal light with differential interference contrast (DIC) microscopy and with epifluorescence, as described in Materials and Methods. GFP localization shows the typical “rim” staining of membrane proteins.

**FIG 6:**
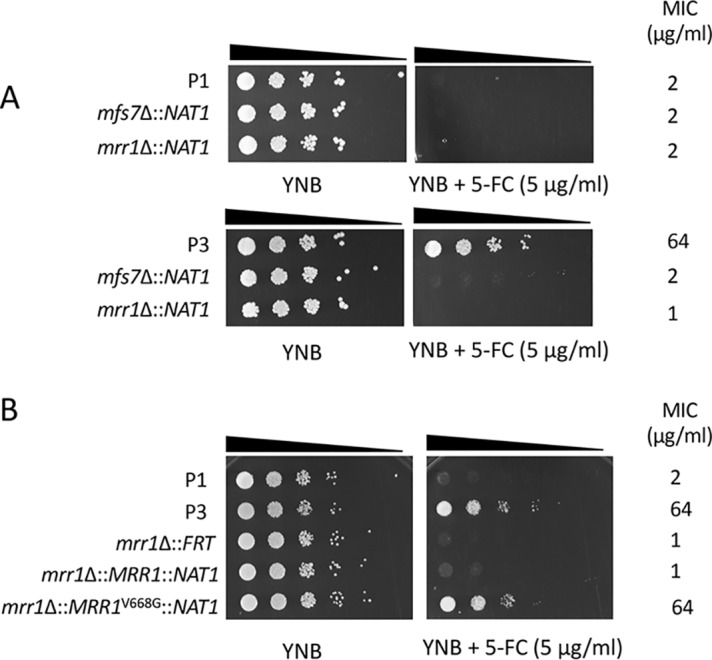
*MRR1* and *MFS7* mediate 5-FC resistance in C. lusitaniae. (A) Tenfold serial dilutions were performed, starting with an inoculum of about 10^5^ cells. Mutants for *MRR1* and *MFS7* in P3 correspond to DSY5240 and DSY5246 and in P1 to isolates DSY5242 and DSY5248, respectively (Table S1). MIC values were obtained by MIC measurements using the SYO system, as described in Materials and Methods. (B) Reversion of *MRR1* deletion. Mutants for *MRR1* correspond to DSY5416. Revertants for the *MRR1* wild-type allele and *MRR1* GOF allele (*MRR1*^V668G^) correspond to isolates DSY5437 and DSY5438, respectively (Table S1).

We showed earlier in the C. lusitaniae isolates recovered from a treated patient that FLC resistance was associated with 5-FC resistance, even if the patient was not exposed to the pyrimidine analog. 5-FC resistance in C. lusitaniae is mediated by mutations occurring in genes involved in 5-FC transport and metabolism (*FCY1* and *FCY2*) (23). In other species, such as C. albicans, other genes (*FUR1*, encoding uracil phosphoribosyltransferase) contain mutations resulting in 5-FC resistance (24). None of these genes contained mutations in isolates P2 to P5. This raised the hypothesis that 5-FC resistance could be mediated by alternative mechanisms, and especially by *MFS7* upregulation, since it results in FLC resistance that itself correlates with 5-FC resistance. We tested this hypothesis by first expressing *MFS7* in a heterologous system in which major C. albicans multidrug transporters (*CDR1*, *CDR2*, *MDR1*, and *FLU1*) were inactivated (25). We observed by serial dilution spotting assays (Fig. 5A) that FLC resistance occurred when *MFS7* was overexpressed, as did the C. albicans ABC transporter *CDR1*, as expected. Interestingly, *MFS7* overexpression resulted in a specific 5-FC resistance since it was not observed by *CDR1* overexpression (Fig. 5A). We also confirmed by green fluorescent protein (GFP) tagging that *MFS7* localized principally to cell structures in C. albicans believed to be cell membranes (Fig. 5B). These data suggest that *MFS7* is a cell membrane-associated transporter for 5-FC. We took advantage of *MRR1* and *MFS7* mutants to address their susceptibility to 5-FC. Figure 6A shows that the deletion of both genes resulted in a 64-fold decrease in the MIC compared to that with the parent strain P3. In addition, the reversion of the *MRR1* deletion by a *MRR1* GOF allele restored 5-FC resistance (Fig. 6B and File S6). Taken together, our data indicate for the first time that *MRR1* is responsible for 5-FC resistance in C. lusitaniae and that this resistance is mediated by *MFS7*, especially when upregulated. This novel mechanism is likely to explain the cross-resistance between the two drug classes in C. lusitaniae.

### *ERG3* and *ERG4* loss of functions and AmB resistance of isolates P2 and P5.

**FIG 7:**
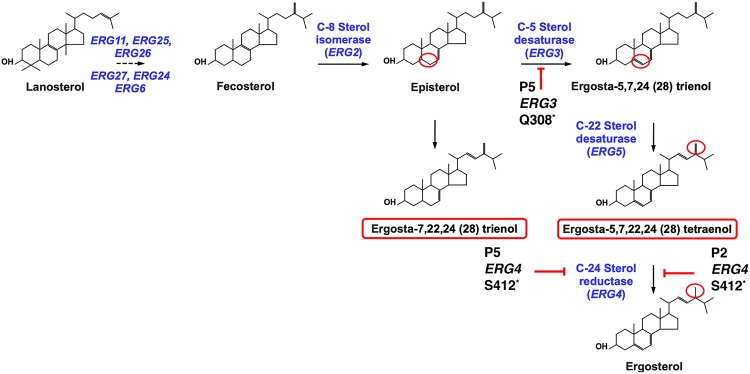
Sterol biosynthesis pathway and defects of isolates P2 and P5. The pathway includes the steps from the substrate lanosterol up to the formation of ergosterol (22). Major genes and sterol intermediates are indicated. *ERG3* and *ERG4* steps are highlighted, and red circles show the activity on the sterol molecule (saturation/desaturation). The *ERG4* defect in P2 and combined defects in *ERG3* and *ERG4* are indicated with corresponding intermediate accumulations (red rectangles).

**FIG 8:**
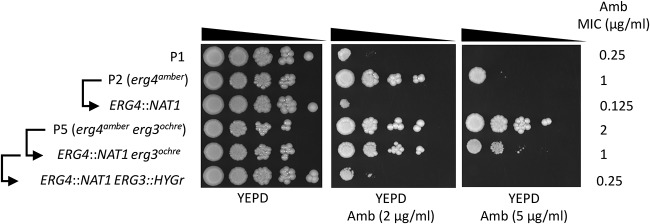
*ERG3* and *ERG4* reversions in isolates P2 and P5. Tenfold serial dilutions were performed, starting with an inoculum of about 10^5^ cells. MIC values were obtained by MIC measurements using the SYO system, as described in Materials and Methods. The *ERG4* revertants from P2 and P5 are DSY5441 (*erg4^amber^*::*ERG4*::*NAT1*) and DSY5444 (*erg4^amber^*::*ERG4*::*NAT1 erg3^ochre^*), respectively. The *ERG4* and *ERG3* revertant from P5 is DSY5452 (*erg4^amber^*::*ERG4*::*NAT1 erg3^ochre^*::*ERG3*::*CaHygB*) (Table S1). Arrows indicate parental relationships between isolates.

In our previous report, the mechanisms behind AmB resistance of isolates P2 and P5 remained puzzling. P2 and P5 isolates exhibited AmB MICs of 1 and 2 μg/ml compared to P1 (MIC, 0.25 μg/ml; File S6). Here, we showed by genome comparisons that the two isolates contained SNPs converting codons of *ERG3* and *ERG4* into stop codons. P2 exhibited a truncation in Erg4p only, while P5 carried truncations in both Erg4p and Erg3p. These mutations could result in loss of function and therefore interruption of ergosterol biosynthesis. Figure 7 shows the sterol biosynthesis pathway and highlights the position of *ERG3* and *ERG4* as well as interference of mutations in P2 and P5 in this pathway. A single *ERG4* loss-of-function mutation is expected to result in the accumulation of ergosta-5,7,22,24-(28)-tetraenol together with other sterol precursors. The combined *ERG3* and *ERG4* loss-of-function mutations are expected to yield ergosta-7,22,24-(28)-trienol as major by-product. These expectations could be verified by mass spectrometry analysis of the sterol fractions of P2 and P5. As shown in Table 4, P2 accumulated ergosta-5,7,22,24-(28)-tetraenol up to 98% in the sterol fraction, while P5 accumulated ergosta-7,22,24-(28)-trienol up to 82% of total sterols with other precursors (fecosterol and episterol) resulting from reactions upstream of *ERG3*. The other AmB-susceptible isolates (P1, P3, and P4) exhibited ergosterol as a major sterol component (95 to 98% of total sterols). Taken together, these sterol analysis suggest that the identified *ERG3* and *ERG4* mutations result in loss of function of the gene products. The accumulation of two separate mutations in P5 yields the expected sterol composition. We believe that the absence of ergosterol in P2 and P5 results in their AmB resistance, as reported earlier (7). To further address the relationships between phenotypes and genotypes in P2 and P5, *ERG3* and *ERG4* wild-type copies were reintroduced in these isolates by a CRISPR-Cas9 approach. Isolate P5 necessitated the restoration of two different wild-type gene copies which could be carried out by the use of two different positive selection markers (*NAT1* and *CaHygB*). Ergosterol biosynthesis was restored in P2/P5 revertants with *ERG3* and *ERG4* wild-type copies (DSY5441 and DSY5452 in Table 4). As shown in Fig. 8, AmB resistance of P2/P5 was reversed to MIC levels of the initial P1 isolate when *ERG3* and *ERG4* wild-type copies were restored in these isolates (MICs, 0.125 and 0.25 μg/ml; File S6). Interestingly, the *ERG4* revertant DSY5444 with the *erg3^ochre^* defective allele still exhibits high AmB MIC (1 μg/ml), which is consistent with absence of ergosterol in this isolate and formation of ergosta-7,22-dienol (Table 4), a known sterol metabolite identified in *erg3 Candida* species mutants (26). The constructed *ERG4* and *ERG3* revertants, while they exhibited changes in AmB MIC values, did not show altered MIC values for other antifungal agents (File S6). This highlights the specificity of the genetic changes and also demonstrates the genetic basis of AmB resistance in isolates P2 and P5.

**TABLE 4:** Sterol composition of isolates P1 to P5 and derived mutants^*a*^

Sterol metabolite	Sterol content of isolates (% mean ± SD)							
	P1	P2 *erg4*^*a*^*^mber^*	P3	P4	P5 *erg4*^*a*^*^mber^ erg3^ochre^*	DSY5441 *ERG4*^*b*^	DSY5444 *ERG4 erg3^ochre^* ^*b*^	DSY5452 *ERG4 ERG3*^*b*^
Ergosta-8,22-dienol							5.8 ± 0.2	
Ergosta-5,8,22,24(28)-tetraenol	0.8 ± 0		0.8 ± 0.1	0.7 ± 0.1		0.3 ± 0.0		0.9 ± 0.3
Ergosta-5,8,22-trienol	0.8 ± 0		0.7 ± 0	0.6 ± 0		0.8 ± 0.0		
Zymosterol	2.2 ± 0.4		2 ± 0.8	2.1 ± 0.1		1.0 ± 0.2		
**Ergosterol**	**95 ± 0.5**		**95.8 ± 0.5**	**94.8 ± 0.3**		**91.3 ± 1.5**	0.3 ± 0.1	**89.2 ± 0.5**
**Ergosta-5,7,22,24(28)-tetraenol**		**98.5 ± 0.8**	0 ± 0.1			5.5 ± 0.9		9.9 ± 0.2
Ergosta-8,22,24(28)-trienol					8.4 ± 0.1			
**Ergosta-7,22-dienol**							**65.8 ± 3.1**	
Fecosterol	0.1 ± 0		0.3 ± 0.2	0.8 ± 0.1	0.8 ± 0.4		2.8 ± 0.6	
**Ergosta-7,22,24(28)-trienol**					**82.3 ± 0.7**	0.6 ± 0.2	11.9 ± 1.1	
Ergosta-5,7-dienol	0.7 ± 0.6			0.3 ± 0.1				
Episterol			0.1 ± 0.2	0.3 ± 0.2	7.5 ± 1.1		7.1 ± 0.9	
Ergosta-7-enol							1.3 ± 0.1	
Lanosterol/obtusifoliol	0.4 ± 0.2		0.2 ± 0.2	0.4 ± 0.2		0.2 ± 0.0		
4,4-dimethyl-cholesta-8,24-dienol						0.3 ± 0.1		
Unknown	0.1 ± 0.1	1.5 ± 1	0.1 ± 0.1		1.1 ± 0.1		2.2 ± 0.2	
Total	100	100	100	100	100	100	97.1	100

a Principal sterols and linked sterol content values are in bold type.

b The complete genotypes can be found in Table S1.

## DISCUSSION

In this work, we showed that the use of genome mining approaches were helpful in the resolution of drug resistance mechanisms in C. lusitaniae. Isolates P1 to P5 were recovered within a 5-month period, and the exploration of genome data revealed a limited spectrum of nucleotides changes between the strains within this time lapse. We obtained five independent chromosome assemblies using the PacBio technology which look essentially similar between isolates P1 to P5, thus indicating the robustness of the assembly approaches. Apart from a specific region duplication and chromosome rearrangements (Chr 6), only a few nonsynonymous SNPs were identified (18) in addition to a higher number of changes in intergenic regions. Comparisons with other available genomes of C. lusitaniae suggested much higher level of nucleotide divergence. In a comparison of coding regions alone between the C. lusitaniae strain ATCC 42720 with P1 to P5, 83,225 SNPs were identified. This accounts for a large nucleotide divergence within the same species, a feature also reported in the study of genome comparisons made in other *Candida* spp. (27, 28). Recently, a study reported the analysis of 20 different C. lusitaniae isolates from a patient with cystic fibrosis (5). The study reported 404 interisolate SNPs (among which 45% were NSS) and 536 indels in total. In comparison, we observed less variation between P1 to P5 in the present work. Additional C. lusitaniae genomes should be analyzed for the further appreciation of genome divergence within this species.

The primary goal of this study was to perform detailed analysis of genomes between isolates P1 to P5 in order to identify the molecular basis of antifungal resistance in the recovered samples. Our data suggest that each isolate originated from the most susceptible isolate, P1. We showed earlier that the specific drug resistance profiles followed the drug regimen (7). The SNP profiles of each isolate now suggest that each isolate underwent independent microevolution trajectories within the host under drug selection. Common SNPs responsible for drug resistance were observed in isolates P3 and P4 (V668G of *MRR1*) and in isolates P2 and P5 (S412* of *ERG4*). This raises the possibility of a closer relationship between these isolate pairs. However, each of the isolates contains additional SNPs and indels which are against the hypothesis of a close lineage, i.e., that each of the P4 and P5 isolates are issued from parents P3 and P2, respectively. Given the number of shared SNPs between the isolates P3/P4 and P2/P5 isolates, it is possible that they originated from common ancestors. More detailed relationships between isolates could have been revealed with a larger isolate collection, however, and this is one of the limitations of our study, that the isolate sampling strategy was not aimed first to address isolate diversity.

FLC resistance was earlier correlated with the upregulation of the multidrug transporter *MFS7*, a close homolog of the C. albicans transporter *MDR1*. Genome comparisons in C. lusitaniae isolates P1 to P5 identified a SNP in *MRR1* leading to a V668G substitution in P3 and P4. We provided genetic evidence (knockouts and reversion experiments) to support the idea that the V668G substitution is a GOF mutation which stimulates *MFS7* expression. These data are consistent with a recent study describing several *MRR1* mutations associated with FLC resistance in C. lusitaniae but not including the V668G GOF mutation (5). This mutation may lead to the upregulation of several other genes, and this hypothesis was confirmed here by RNA-seq analysis of the P3 and P4 isolates. Interestingly, our data match well with those comparing an azole-resistant isolate with an azole-susceptible C. lusitaniae isolate (5). From the 19 identified regulated genes in this work (5), 12 were corresponding in our data set, and *MFS7* (also called *MDR1*) was among the upregulated genes in the azole-resistant isolates (File S4).

It was interesting to observe that deletions of *MRR1* and *MFS7* in P3 resulted in different FLC MIC values (MICs, 1 μg/ml and 8 μg/ml, respectively). This underscores that, in addition to *MFS7*, *MRR1* controls other FLC resistance mediators. One likely candidate could be the *CDR1* homolog EJF14_30164, which is 2- to 4-fold upregulated in P3 and P4, respectively, compared to P1 (File S4). *CDR1*, also known as ABC15 (29), was shown previously as slightly upregulated in P3 and P4 compared to P1 (7). *CDR1* and homologs are known to mediate azole efflux and are important contributor of azole resistance in several fungal pathogens (30). *CDR1* may be controlled by *MRR1* in C. lusitaniae. This contrasts with the regulatory properties of *MRR1* in C. albicans, which do not include *CDR1* (31). Further work is needed to address this question.

The *MRR1* V668G substitution was associated with another phenotype (resistance to 5-FC), and we propose that the multidrug transporter *MFS7* (a major facilitator efflux transporter) could mediate this phenotype. The data supporting this conclusion are based on genetic approaches as well as *MFS7* overexpression in C. albicans. Until now, 5-FC resistance in *Candida* spp. has been looked essentially at the angle of drug import, and our work provides evidence that drug efflux may also be involved. One can also argue that the known 5-FC import system (such as *FCY2*) could be regulated in the presence of the *MRR1* V668G mutation, which would result in 5-FC resistance; however, our RNA-seq data do not support this hypothesis (data not shown). Interestingly, it was shown in C. lusitaniae by Noël et al. (4) that FLC and 5-FC can exhibit cross-resistance when the two drugs are combined *in vitro*; however, the mechanism behind this observation remained elusive. In this experimental setting, we propose that *MFS7* could have been upregulated in a transient manner by the addition of both drugs *in vitro*. This possible elevated expression could be sufficient to result in a cross-resistance phenotype. Additional work is warranted to support this hypothesis.

The genome comparisons out of PacBio assemblies identified the same three separate *FKS1* mutations that were earlier reported in isolates P2, P4, and P5 (7). We also confirmed in our first study that the *FKS1* amino acid substitutions S638P, S638Y, and S631Y were causing candin resistance, using S. cerevisiae as a surrogate (7). Recently, it was reported that S638P substitution could mediate candin resistance in C. lusitaniae. This was achieved by allelic replacement in this species (32) and thus confirms the importance of the *FKS1* position 638^Ser^ in establishing candin resistance in C. lusitaniae. The Fks1p position 638^Ser^ lies within a region called hot spot region 1 (HS1) that is enriched in amino acid substitutions responsible for candin resistance (32). Position 631^Ser^ is the only position that was yet not been confirmed as causing candin resistance in C. lusitaniae; however, its proximity to HS1 and our own study with S. cerevisiae makes the S631Y substitution a likely cause of candin resistance.

AmB resistance in fungal pathogens is usually caused by mutations in the ergosterol biosynthesis pathway (33). C. lusitaniae develops resistance to AmB during infections and also by inactivation of specific *ERG* genes *in vitro* (34). In this work, we provide evidence that AmB resistance is mediated by the loss of function of two genes, *ERG3* and *ERG4. ERG3* loss-of-function mutations have been documented in several fungal pathogens, all resulting in AmB resistance (26, 35–38). To our knowledge, *ERG4* loss-of-function mutations have not been described so far in fungal pathogens. We show here that *ERG4* loss of function results in the absence of ergosterol, thus contributing to AmB resistance, as measured earlier (7). A surprising result was the combination of the two loss-of-function mutations in isolate P5, which was the last recovered isolate from the treated patient. The accumulation of two different loss-of-function *ERG* mutations in the same isolate is unique, to our knowledge, in fungal pathogens. Until now, the combination of *ERG* mutations was only known in C. albicans for functional *ERG11* point mutations combined with defects in *ERG5* or *ERG3* leading to both azole and AmB resistance (35, 39). Interestingly, isolate P5 exhibits a slightly higher AmB MIC than does P2 (2 versus 1 μg/ml, respectively; File S6), and thus, it is likely that the accumulation of two *ERG* mutations conferred enhanced protection against the activity of AmB in this isolate. Supposing that P2 is the parent of P5, this implies that the *ERG3* mutation occurred sequentially after the emergence of the *ERG4* mutation; however, we cannot firmly confirm this hypothesis. The combination of the loss-of-function mutations, irrespective of their timely occurrence, rather suggests functional compensatory effects. For example, one of the mutations in the *ERG* genes (i.e., *ERG4*) could have resulted in fitness defects and therefore decreased virulence. We addressed this hypothesis by testing the virulence of each isolate in the minihost Galleria mellonella. Our results could not detect significant virulence differences between strains (Fig. S2). The necessity for P5 to carry two independent *ERG3* and *ERG4* loss of function may be therefore solely explained by the resulting benefit in AmB resistance.

10.1128/mBio.02512-19.2 FIG S2 Galleria mellonella infection with isolates P1 to P5. Data were plotted with Prism 8.1.2 (GraphPad Software, San Diego, CA, USA), and no statistical differences were noticed between the survival rates of the tested isolates. Download FIG S2, PDF file, 0.1 MB. Copyright © 2019 Kannan et al. 2019 Kannan et al. This content is distributed under the terms of the Creative Commons Attribution 4.0 International license.

Taken together, the results of our study highlight that genome comparisons are highly relevant in the resolution of drug resistance mechanisms. Genome sequencing is becoming affordable, and several other studies have already taken comparative genomics as a way to detect antifungal resistance mechanisms (40). Nevertheless, the relevance of genome alterations needs to be addressed by parallel experimental approaches that are now greatly facilitated by the development of novel genome editing technologies (such as the use of CRISPR-Cas9).

## MATERIALS AND METHODS

### Strains and media.

C. lusitaniae and C. albicans isolates were grown in YEPD complete medium (1% Bacto peptone; Difco Laboratories, Basel, Switzerland), 0.5% yeast extract (Difco), and 2% glucose (Fluka, Buchs, Switzerland) at 30°C under agitation. The genotypes of all strains constructed are listed in Table S1. Plasmids were propagated in Escherichia coli DH5α on Luria-Bertani (LB) 2% agar plates at 37°C overnight (41). LB medium was supplemented with either 100 μg/ml ampicillin (AppliChem) or 34 μg/ml chloramphenicol (Fluka) when necessary.

### Antifungal susceptibility testing.

Antifungal susceptibility testing was carried out using the Sensititre YeastOne (SYO) colorimetric microdilution method and was performed using commercially available panels (Thermo Fisher Scientific, Switzerland), according to the manufacturer’s recommendation. The serial dilution susceptibility method was performed onto agar plates with either YEPD or YNB minimal medium (0.67% yeast nitrogen base [Difco] with 2% glucose). Yeast cells from an overnight culture in YEPD medium were diluted to 1.5 × 10^7^ cells/ml in 1 ml phosphate-buffered saline (PBS; Bicshel, Interlaken, Switzerland), and 200 μl of this solution was transferred to a 96-well flat-bottom plate (Sigma-Aldrich). Tenfold serial dilutions were performed from 1.5 × 10^7^ to 1.5 × 10^2^ cells/ml in PBS. The cell dilutions were next spotted on plates with a 48-pin replicator (V&P Scientific, Inc., San Diego, CA, USA) and the plates incubated at 35°C for 24 to 48 h.

### PacBio genome sequencing.

In order to produce high-quality genomic DNA from C. lusitaniae isolates, overnight cultures (5 ml) were grown in YNB minimal medium (0.67% yeast nitrogen base [Difco] with 2% glucose) at 30°C to obtain 2 × 10^8^ cells for each strain. DNA isolation followed the instructions of the Gentra Puregene Yeast/Bact kit (Qiagen), with slight modifications. First, yeast cell lysis was performed with Zymolyase 100T (3 μg/μl end concentration) for 30 min at 37°C. In addition, phenol-chloroform extractions were carried out after RNase A treatment of precipitated nucleic acids, according to recommendations issued by Pacific Biosciences (PacBio SampleNet-shared protocol) for the use of phenol-chloroform-isoamyl alcohol. After final precipitation with NH_4_OAc and several washes with 80% alcohol, the genomic DNA was dissolved carefully in a small volume of elution buffer (10 mM Tris-HCl [pH 8.5]). Aliquots of 5 μg of extracted, high-quality, genomic DNA was diluted to 150 μl using elution buffer at 30 μg/μl. Long-insert SMRTbell template libraries were prepared (20-kb insert size) according to PacBio protocols. Each isolate used 2 single-molecule real-time (SMRT) cells, which were sequenced using P6 polymerase binding and C4 BluePippin sequencing kits with 240-min acquisition time on PacBio RSII at the Lausanne Genomic Technologies Facility (LGTF) of the University of Lausanne (Unil). *De novo* genome assemblies were produced using PacBio’s SMRT Portal (v2.3.0) and the Hierarchical Genome Assembly Process (HGAP version 3.0), with default settings and a seed read cutoff length of 6,000 bp.

### Annotation.

For the C. lusitaniae annotation process, we took advantage of available RNA-seq reads obtained from isolates P1, P3, and P4 isolates (see below). These reads were used to predict and confirm CDS of the sequenced genomes. Processed reads were aligned against the P1 reference genome available as a fasta file from PacBio assemblies (see below), and the resulting alignments were assembled into potential transcripts using StringTie (v1.3.3) (42). These transcript assemblies were subsequently used as expressed sequence tag (EST) evidence for assigning the structural annotations to the P1 genome using Work-Queue Maker (WQ-MAKER) (43). The UniProt C. lusitaniae protein data set was downloaded at https://www.uniprot.org/uniprot/?query=candida+lusitaniae&sort=score. These protein sequences were used as protein evidence during structural annotations. We used MAKER (44) that incorporates SNAP (45) into the gene prediction pipeline to annotate the P1 genome assembly. SNAP gene predictions make use of hidden Markov models (HMMs) as their underlying probabilistic model. Repetitive elements, including low-complexity sequences and interspersed repeats in the input genome sequence, were identified and masked using RepeatMasker (46) by aligning the nonannotated genome against a library of known repeats, such as Repbase (47). Transcript assemblies and protein sequences described above were used as evidence to aid gene predictions. In the initial run, MAKER was launched iteratively, and the tasks such as repeat masking and evidence alignments were performed, which resulted in a general feature format 3 (GFF3) file containing the masked regions and protein transcript alignments. The GFF3 file generated from the above-mentioned step was used during subsequent MAKER runs. The data generated in the initial run were used in training the gene predictions using SNAP. All the transcript sequences that were used as evidence during the initial MAKER run were placed into a single transcript fasta file and were used for SNAP HMM training. After training SNAP HMMs from iterative runs that generate imperfect gene models, MAKER was run once again in the final step to accurately predict genes and corresponding ORFs.

Following the detection of ORFs, all proteins of the P1 genome were subjected to a BLAST search to the Swiss-Prot database (release-2017_09) using Blast2GO (version 5.2.5; BioBam Bioinformatics, Valencia, Spain). Top hits were retrieved from the BLAST results (E value cutoff, 10^−3^) and were added to each ORF annotation. ORFs without BLAST results were resubmitted using Blast2GO to the nonredundant protein NCBI database to enlarge the BLAST search, and positive results were added to the existing annotations.

### Genome comparisons.

Annotated genomes were compared using Mauve (version 2015-02-25), with default parameters, and the aligner software Muscle 3.6. The produced alignments were next imported in Geneious Prime (2019.1.3; Biomatters, Ltd., Auckland, New Zealand), and each chromosome alignment was exported as a nucleotide alignment file. After establishing genome of isolate P1 as a reference, SNPs and insertions/deletions were obtained by the software by selection of coding and noncoding regions.

SNP densities along chromosomes were obtained from VCF files extracted from each chromosome comparison using Geneious. VCF files corresponding to each chromosome were imported into the online software SNiPlay (48) with a size of the sliding window of 2,000 nucleotides. Data were exported in Prism 8.1.2 (GraphPad Software, San Diego, CA, USA) for visualization.

For P1 to P5 genome comparisons, differences between genomes in homopolymeric regions [poly(dN) stretches of at least 7 nucleotides] were ignored since they were likely due to artifacts of the PacBio sequencing technology, as reported earlier (49). Only differences in these stretches occurring in at least 2 separate genomes were considered in genome comparisons. In addition, only telomeric chromosome regions that were common to all P1 to P5 isolates were included in genome comparisons.

### Genome-wide transcriptional analysis.

**(i) RNA extraction and processing.** RNA was isolated from 5-ml log-phase cultures of isolates P1, P3, and P4 grown in YEPD medium at 30°C under agitation. Total RNA was extracted from with the RNeasy Protect minikit (Qiagen) by a process involving mechanical disruption of the cells with glass beads, as previously described (50). Total RNA extracts were treated with DNase using a DNA-free kit (Ambion-Life Technologies, Zug, Switzerland). RNA quality and integrity were verified with a fragment analyzer automated capillary electrophoresis (CE) system (Advanced Analytical). RNA extractions were performed in biological triplicate. RNA libraries for RNA-seq were prepared with a TruSeq stranded total RNA library prep kit (Illumina). The resulting libraries were sequenced on an Illumina HiSeq 2500 system at the LGTF.

**(ii) Quality control and processing of raw RNA-seq reads.** Twenty-seven single-end RNA-seq data sets from three different C. lusitaniae strains (P1, P3, and P4) after RNA sequencing were obtained. The quality of the raw reads was assessed using FastQC (v0.11.7) (51). Prior to mapping and assembly, adapter trimming, quality filtering, artefact removal, and contaminant filtering were carried out with BBDuk (v38.51) from the BBTools package (52). Low-complexity filtering and removal of rRNA sequences were carried out using Prinseq (v0.20.3) (53) and SortMeRNA (v2.1) (54), respectively. The processed reads were aligned to the P1 annotated genome using HSAT2 (v2.1) (55).

**(iii) Differential gene expression analysis.** Data normalization and differential expression analysis were performed in R (v3.5.2) using the Bioconductor packages. The read count data were normalized with the TMM (trimmed mean of M-values) method available in the R Bioconductor package edgeR (56) and subsequently transformed to log_2_ counts per million by voom, a method implemented in the R Bioconductor package limma (57). A linear model with one factor per condition was applied to the transformed data using limma (58).

### *MRR1*/*MFS7* gene deletions.

**(i) Plasmid constructions.** In order to delete *MRR1* and *MFS7* in C. lusitaniae, the flanking regions of the two genes were cloned by PCR into pSFS2A (59). *MRR1* flanking regions were amplified from isolate P1 with the primer pairs ClMRR1-Apa/ClMRR1-Xho and ClMRR-SacI/ClMRR-SacII (Table S1). *MFS7* flanking regions were amplified from isolate P1 with the primer pairs MFS7-Kpn/MSF7-Xho and MFS7-SacI/MFS7-SacII (Table S1). PCR products were cloned sequentially into corresponding sites to result in plasmid pDS1860 (*MFS7* inactivation) and pDS1864 (*MRR1* inactivation). pDS1860 and pDS1864 were next modified by removing the *SAT1* flipper cassette system by BamHI/NotI digestion and replacement with the *NAT1* dominant marker amplified from pJK795 (60) using primers NAT1-BglII and NAT1-Not, thus resulting in pDS2039 and pSD2038, respectively.

**(ii) Cas9-CRISPR for knockout of *MRR1* and *MFS7*.** The RNA-protein complex (RNP) approach reported by Grahl et al. (61) was used that employs reconstituted purified Cas9 protein in complex with scaffold and gene-specific guide RNAs. Genomic RNA (gRNA) specific for *MRR1* and *MFS7* (Table S1) were selected and obtained from Integrated DNA Technologies, Inc. (IDT) as CRISPR guide RNA (crRNA), which contains 20 bp homologous to the target gene fused to the scaffold sequence. Gene-specific RNA guides were designed *in silico* using Geneious Prime. RNPs were created using the Alt-R CRISPR-Cas9 system from IDT. Briefly, crRNAs (crMRR1 and crMFS7, Table S1) and tracrRNA (a universal transactivating CRISPR RNA) were first dissolved in RNase-free distilled water (dH_2_O) at 100 μM and stored at –80°C. The complete guide RNA was generated by mixing equimolar concentrations (4 μM final) of the gene-specific crRNA and tracrRNA to obtain a final volume of 3.6 μl per transformation. The mix was incubated at 95°C for 5 min and cool down to room temperature. The Cas9 nuclease 3NLS (60 μM stock from IDT) was diluted to 4 μM in dH_2_O at a volume of 3 μl per transformation. RNPs were assembled by mixing guide RNAs (3.6 μl of gene-specific crRNA/tracrRNA) with 3 μl of diluted Cas9 protein, followed by incubation at room temperature for 5 min. Transformation of C. lusitaniae cells was carried out by electroporation and used 6.6 μl of gene-specific RNPs, 40 μl of C. lusitaniae cells, and 1 to 2 μg of repair constructs (up to 3.4 μl volume). Repair constructs containing the *MRR1* and *MFS7* inactivation cassettes were obtained by PCR amplification with primer pairs ClMRR1-Apa/ClMRR-SacI and MFS7-Kpn/MFS7-SacI from pDS2038 and pSD2039, respectively. Transformants were selected at 30°C on YEPD agar containing 200 μg/ml nourseothricin. Transformants were verified by PCR using the primer pair NAT1_134_R/ClMRR1-verif3 for *MRR1* deletion and NAT1_134_R/5-MFS7-A for *MFS7* deletion. DNA from transformants was prepared by small-scale rapid DNA extraction, as described previously (62).

**(iii) *MRR1* reversion.** In order to reintroduce *MRR1* alleles in the background of *MRR1* deletion mutants, an alternative mutant construction using a recyclable *NAT1* marker was employed. The maltose-inducible *MAL2*-*FLP1* system of pSFS2A was first excised from pSFS2A as a 0.9-kb ApaI-EcoRV fragment and cloned into pJK863 to substitute the *SAP2* promoter, thus resulting in pDS2046. In this approach, the *NAT1* marker could be recycled by *MAL2*-dependent *FLP1* expression (MAL2-FLP-NAT1). This plasmid was used as the PCR template with the primer pair MRR1-5_pDS2046/MRR1-3_pDS2046. Both primers contained 70-bp homology to the *MRR1* 5′- and 3′-flanking regions and a 21-bp extension matching to the MAL2-FLP-NAT1 extremities. CRISPR-Cas9-mediated recombinations at *MRR1* flanking regions with this PCR-amplified repair fragment were used with crRNAs crMRR1_del5 and crMRR1_del3 that were prepared as explained above to reconstitute functional RNPs, with the exception that both RNPs were concentrated by 2-fold. Transformation of C. lusitaniae was carried out by electroporation, as described below, and transformants were selected onto YEPD plates with nourseothricin (200 μg/ml).

After *MRR1* deletion verification by PCR, as described above, strains were grown overnight on YEP liquid medium with 2% maltose at 30°C in order to induce *FLP1*-mediated recombination at FLP recombination target (FRT) sequences and the resulting loss of *NAT1*. Recycling of *NAT1* was observed in YEPD agar medium containing each about 10^2^ C. lusitaniae cells at a nourseothricin concentration of 1 μg/ml to distinguish between parent cells and those without *NAT1*.

Nourseothricin-sensitive C. lusitaniae cells deleted for *MRR1* were used for *MRR1* reversion. *MRR1* alleles were first cloned into pSD2038 with fragments obtained by PCR using primers ClMRR1-Apa and ClMRR1-Xhorev and DNA templates from isolates P1 and P3, which resulted in plasmids pDS2040 and pDS2041, respectively. Repair fragments were obtained from both plasmids with primers ClMRR1-Apa and MRR1-3_rev_new. CRISPR-Cas9-mediated recombinations at the *MRR1* flanking regions with these PCR-amplified repair fragments were used with crRNAs crRNA_MRR1_rev5 and crRNA_MRR1_rev3 that were prepared as described above to reconstitute functional RNPs. Transformations of C. lusitaniae were carried out by electroporation, as described below, and transformants were selected onto YEPD plates with nourseothricin (200 μg/ml). Reintegration of *MRR1* alleles was verified by PCR on recovered genomic DNA with the primer pair ClMRR1_F/ClMRR1_3377_R, followed by sequencing with primer ClMRR1_2900_F to confirm allele identity.

### *MFS7*-GFP tagging.

GFP tagging of *MFS7* was realized by a Gateway cloning approach developed by Chauvel et al. (63). *MFS7* was first amplified with the primers Forward_gateway_MFS7 and Reverse_gateway_MFS7 (Table S1) and cloned into pDONR207 (63) by a recombination reaction with Invitrogen Gateway BP Clonase. Next, *MFS7* was transferred into pCA-DEST1102 (a plasmid aimed to express C-terminal GFP fusion proteins under the control of a tetracycline-regulated promoter) by LR Clonase resulting in pDS2034. Next, this plasmid was linearized by i-SceI and transformed for Ura^+^ selection in strain CPY41, a derivative of SC5314 containing pNIMX (63) and lacking *URA3* alleles, to yield DSY5194.

### *MFS7* overexpression.

*MFS7* overexpression used a system that overexpresses proteins in C. albicans through a *CDR1* promoter under the control of a *TAC1* gain-of-function allele (25). The *MFS7* ORF was amplified from isolate P1 with primers MFS7_XbaI and MFS7_Nhe and cloned into the single SpeI site of pDS1873, a derivative of pDS1874 lacking *CDR1-GFP* (25), to yield pDS2022. This plasmid was digested by SacI and PvuI to allow recombination at the *CDR1* locus and transformed into DSY4684 lacking major multidrug transporters (25). As control, DSY4684 was transformed with CIp10 after StuI digestion (64). Transformants were obtained by Ura^+^ selection.

### *ERG3* and *ERG4* reversions.

In order to restore wild-type copies of *ERG3* and *ERG4* in isolates with defects in these genes, a CRISPR approach was used. First, repair fragments were constructed by fusion PCR. The *ERG4* repair fragment was generated by fusion of three PCR fragments with overlapping sequences. The first PCR fragment amplified the wild-type *ERG4* from isolate P1 with primers ERG4-P1 and ERG4-NAT1-R (20-bp overlap with pJK795 [60]). The second PCR fragment amplified the selective marker *NAT1* from pJK795 with primers ERG4-NAT1-F (20-bp overlap with *ERG4*) and ERG4-NAT1t-R (20-bp overlap with pJK795). The third PCR fragment amplified a 3′-end portion of *ERG4* with primers ERG4-P2 and ERG4-NAT1t-F (20-bp overlap with pJK795). The final PCR was performed with the 3 purified fragments and nested primers ERG4-P3 and ERG4-P4 in the presence of 1.2 M betaine. The *ERG3* repair fragment was constructed in a similar fashion but using the hygromycin resistance selection marker from pYM70 (65). The first PCR fragment amplified the wild-type *ERG3* from isolate P1 with primers ERG3-P1 and ERG3-HYG-R (20-bp overlap with pYM70). The second PCR fragment amplified the selective marker *CaHygB* from pYM70 with primers ERG3-HYG-F (20-bp overlap with *ERG3*) and ERG3-HYGt-R (20-bp overlap with pYM70). The third PCR fragment amplified a 3′-end portion of *ERG3* with primers ERG3-P2 and ERG3-HYGt-F (20-bp overlap with pJK795). The final PCR was performed with the 3 purified fragments and nested primers ERG3-P3 and ERG3-P4 in the presence of 1.2 M betaine.

RNPs were reconstituted as described above. gRNA specific for *ERG3* and *ERG4* (Table S1) was selected for targeting the mutated positions in isolates P2 and P5 and were obtained from Integrated DNA Technologies, Inc. (IDT) as CRISPR guide RNA (crRNA) fused to the scaffold sequence. RNPs were assembled by mixing guide RNAs (3.6 μl of gene-specific crRNA/tracrRNA) with 3 μl of diluted Cas9 protein, followed by incubation at room temperature for 5 min. Transformation of C. lusitaniae cells was carried out by electroporation as described below and used 6.6 μl of gene-specific RNPs, 40 μl of C. lusitaniae cells, and 1 to 2 μg of *ERG3* or *ERG4* repair constructs (up to 3.4 μl volume). Isolates P2 and P5 were used with the *ERG4* repair fragment, and transformant selection was operated onto nourseothricin-selective medium (200 μg/ml nourseothricin). The *ERG3* repair fragment was used with an *ERG4* revertant from P5 and transformant selection was operated on hygromycin-selective medium (250 μg/ml hygromycin).

Verification of *ERG3* and *ERG4* reversions was carried out by PCR with primers ClERG4_1427_F and NAT1_134_R (*ERG4*) and primers ClERG3_1104_F and ERG3-HYG-R (*ERG3*), followed by sequencing.

### C. lusitaniae transformation.

Transformations in C. lusitaniae were carried out by electroporation. Overnight-grown cells were freshly diluted 20-fold in 20 ml YEPD medium and grown to logarithmic phase to a density of 1 × 10^7^ to 2 × 10^7^ cells/ml at 30°C under constant agitation. After centrifugation for 5 min at 2,500 rpm at 4°C and removal of the supernatant, cells were resuspended in 10 ml transformation buffer (100 mM lithium acetate [LiAc], 100 mM dithiothreitol [DTT], 10 mM Tris-HCl [pH 7.5], 1 mM EDTA) and incubated for 1 h at room temperature under mild shaking. Cells were next washed twice with ice-cold water and with 1 M sorbitol. Cell pellets were finally resuspended in 200 μl 1 M sorbitol. Transformations were carried out with aliquots of 40 μl of slurry in 0.2-cm electroporation cuvettes (Bio-Rad) with the following settings: 1.8 kV, 200 Ω, and 25 μF. After an electroporation pulse, 1 ml YEPD was immediately added, and cells were incubated overnight with gentle shaking at 30°C. Once the cells were allowed to recover overnight at room temperature, aliquots were plated onto the corresponding selective media.

### Quantitative reverse transcription-PCR.

Total RNA was extracted as described above from log-phase cultures grown in YEPD at 30°C under constant agitation. Gene expression levels were determined by real-time quantitative reverse transcription-PCR (qRT-PCR) in a StepOne real-time PCR system (Applied Biosystems) using the Mesa Blue qPCR MasterMix Plus for SYBR assay kit (Eurogentec). Each reaction was performed in triplicate on three separate occasions. Expression levels of *MFS7* were normalized by *ACT1* expression, as described previously (7).

### Sterol analysis.

Overnight cultures of C. lusitaniae strains were used to inoculate 10 ml YEPD at a starting concentration of 1 × 10^4^ cells/ml. Cultures were grown for 18 h at 37°C and 200 rpm. Cells were then harvested and pellets washed twice with double-distilled water (ddH_2_O). Sterols were extracted and derivatized as previously described (66). Briefly, lipids were saponified using alcoholic KOH and nonsaponifiable lipids extracted with hexane. Samples were dried in a vacuum centrifuge and were derivatized by the addition of 0.1 ml BSTFA [*N*,*O*-Bis(trimethylsilyl)trifluoroacetamide] and trimethylchlorosilane (TMCS) (99:1; Sigma) and 0.3 ml anhydrous pyridine (Sigma) and heated at 80°C for 2 h. TMS-derivatized sterols were analyzed and identified using gas chromatography-mass spectrometry (GC/MS) (Thermo 1300 GC coupled to a Thermo ISQ mass spectrometer; Thermo Scientific) and the Xcalibur software (Thermo Scientific). The retention times and fragmentation spectra for known standards were used to identify sterols.

### Virulence assays with Galleria mellonella.

Galleria mellonella larvae were purchased from TruLarv (Biosystems Technology, Exeter, Devon, UK). Larvae weighing between 0.2 and 0.3 g were selected for our experiments and stored at 16°C upon arrival. A total of 10 larvae were infected with each isolate by microinjection (Omnican100; BBraun). C. lusitaniae cells were grown overnight in YEPD medium at 30°C under agitation and pelleted by centrifugation (5 min at 4,600 rpm). Cells were washed twice with phosphate-buffered saline (PBS; 137 mM NaCl, 10 mM phosphate, 2.7 mM KCl [pH 7.4]), and the amount of cells was estimated with a photometer (Novaspec II; Pharmacia). Forty microliters of a cell suspension containing 1.5 × 10^7^ cells/ml in PBS was then injected into the last left proleg. Control larvae were injected with the same volume of PBS. The injected larvae were incubated at 30°C in the dark for 9 days postinfection, and survival was scored each day. Larvae were scored as dead when melanized and upon lack of response after gentle manipulation with a clamp.

### Microscopy.

Epifluorescence and phase-contrast imaging were performed with a Zeiss Axioplan 2 microscope equipped for epifluorescence microscopy with a 100-W mercury high-pressure bulb and Zeiss filter set 9 (for GFP imaging). Images obtained with a SPOT RT3 cooled 2 Mp charge-coupled-device (CCD) camera (Diagnostic Instruments, Inc., MI, USA) were recorded and captured with VisiView (Visitron Systems GmbH, Germany).

### Data availability.

Strains described here are available upon request. PacBio assemblies are available under BioProject number PRJNA504391. RNA-seq data are available under study number SRP172837. The final assembled genomes for isolates P1 to P5 and their annotations are available under accession numbers CP038484 to CP038491, CP039550 to CP039557, CP039652 to CP039659, CP039618 to CP039625, and CP039660 to CP039667, respectively.
