# Erratum for Kannan et al., “Comparative Genomics for the Elucidation of Multidrug Resistance in Candida lusitaniae*”*

**DOI:** 10.1128/mBio.03403-19

**Published:** 2020-02-11

**Authors:** Abhilash Kannan, Sandra A. Asner, Emilie Trachsel, Steve Kelly, Josie Parker, Dominique Sanglard

**Affiliations:** a Institute of Microbiology, Department of Laboratory, Lausanne University Hospital, Lausanne, Switzerland; b Unit of Pediatric Infectious Diseases and Vaccinology, Department of Paediatrics, Lausanne University Hospital, Lausanne, Switzerland; c Infectious Diseases Service, Department of Internal Medicine, Lausanne University Hospital, Lausanne, Switzerland; d Institute of Life Science, Swansea University Medical School, Swansea, United Kingdom

## ERRATUM

Volume 10, issue 6, e02512-19, 2019, https://10.1128/mBio.02512-19. In Results, Fig. 3 (PDF page 9) was erroneously duplicated from Fig. 6 at the proofreading stage and should be replaced by the Fig. 3 included in this erratum, with the caption corrected for two strain designation numbers (DSY5246 and DSY5242). The correct Fig. 3 in this erratum was in the original accepted version of the manuscript. This erratum does not change the major scientific conclusions of the work.

**FIG 3:**
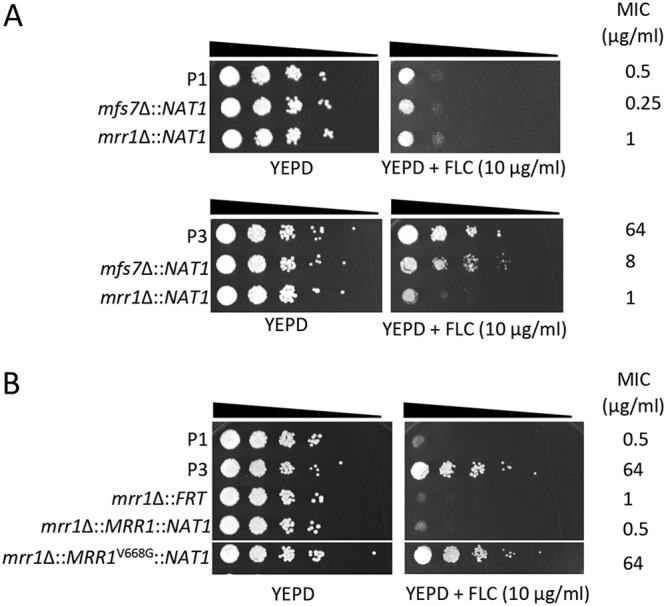
*MRR1* and *MFS7* mediate FLC resistance in C. lusitaniae. (A) Tenfold serial dilutions were performed, starting with an inoculum of about 10^5^ cells. Mutants for *MRR1* and *MFS7* in P3 correspond to DSY5240 and DSY5246 and in P1 to isolates DSY5242 and DSY5248, respectively. MIC values were obtained by MIC measurements using the SYO system, as described in Materials and Methods. (B) Reversion of *MRR1* deletion. Mutants for *MRR1* correspond to DSY5416. Revertants for the *MRR1* wild-type allele and *MRR1* GOF allele (*MRR1*^V668G^) correspond to isolates DSY5437 and DSY5438, respectively (Table S1). The white line indicates removal of a yeast sample from the original agar plate.

