# Supplementary material for: Comparative Genomics for the Elucidation of Multidrug Resistance in Candida lusitaniae
Source: mBio. 2019 Dec 24;10(6):e02512-19. doi: 10.1128/mBio.02512-19 (PMC6935856; doi:10.1128/mBio.02512-19)
Supplement: TABLE S2 [file mBio.02512-19-st002.docx]

**Table S3: Chromosome lengths of *C. lusitianiae* chomosomes**

| Isolate | Chr 1 | Chr 2 | Chr 3 | Chr 4 | Chr 5 | Chr 6 | Chr 7 | Chr 8 | Total nucleotides |
| --- | --- | --- | --- | --- | --- | --- | --- | --- | --- |
| DSY4606 | 2445101^a^ | 2009500 | 2006239 | 1662877 | 1481603 | 1039973 | 744510 | 692029 | 12081832 |
| DSY4593 | 2445049 | 2009583 | 2006033 | 1708559 | 1467690 | 1030235 | 744525 | 692229 | 12103903 |
| DSY4662 | 2445445 | 2009609 | 2006152 | 1702157 | 1465816 | 1026012 | 744434 | 687269 | 12086894 |
| DSY4590 | 2445561 | 2009381 | 2006156 | 1673417 | 1482565 | 1039049 | 744570 | 687337 | 12088036 |
| DSY4661 | 2445479 | 2009583 | 2006234 | 1651558 | 1487923 | 1059187 | 744370 | 692312 | 12096646 |

^a^: Number of nucleotides
