# Supplementary figures and images for: Comparative Genomics for the Elucidation of Multidrug Resistance in Candida lusitaniae
Source: mBio. 2019 Dec 24;10(6):e02512-19. doi: 10.1128/mBio.02512-19 (PMC6935856; doi:10.1128/mBio.02512-19)

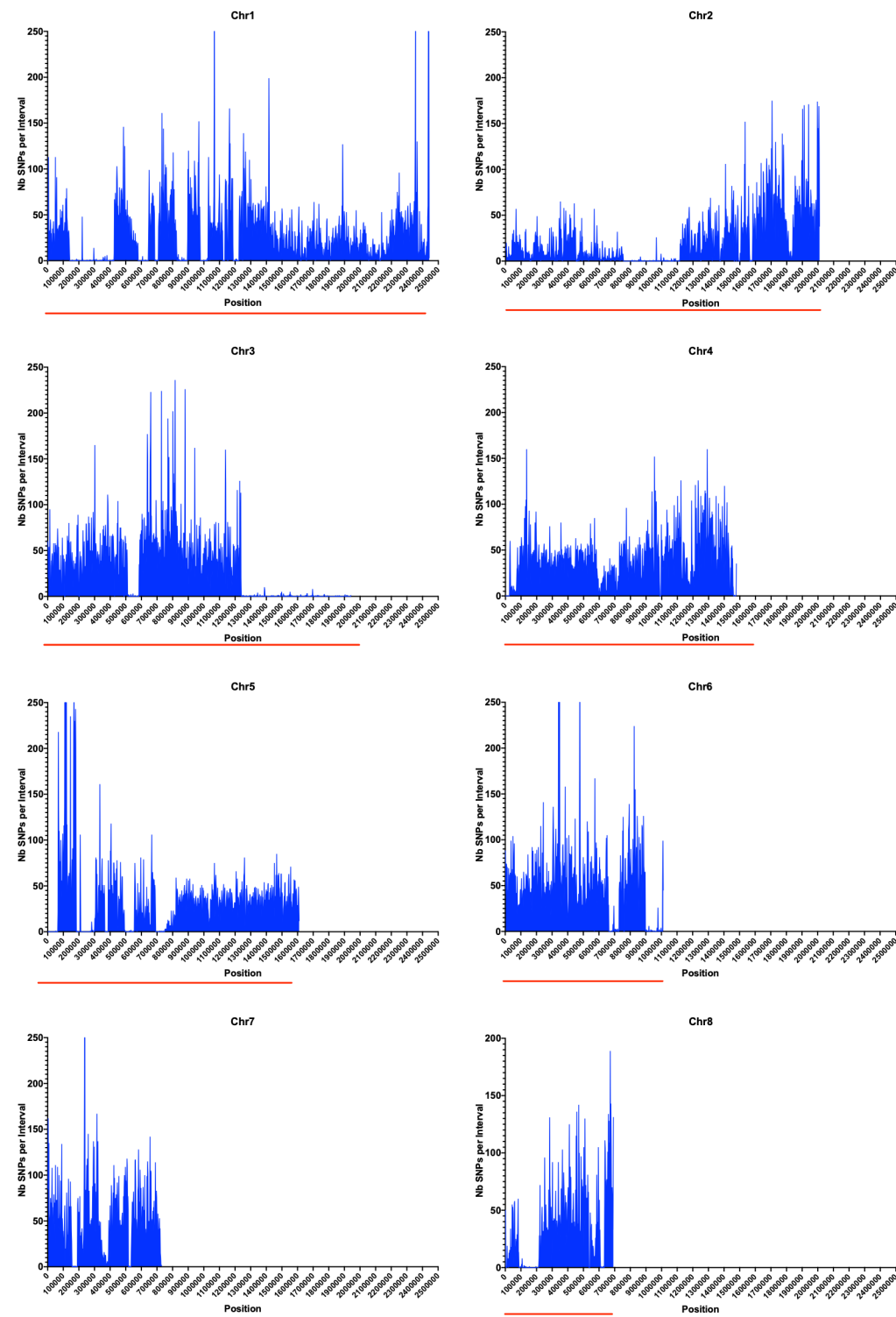

Fig S1

Supplement: FIG S1 [file mBio.02512-19-sf001.pdf]

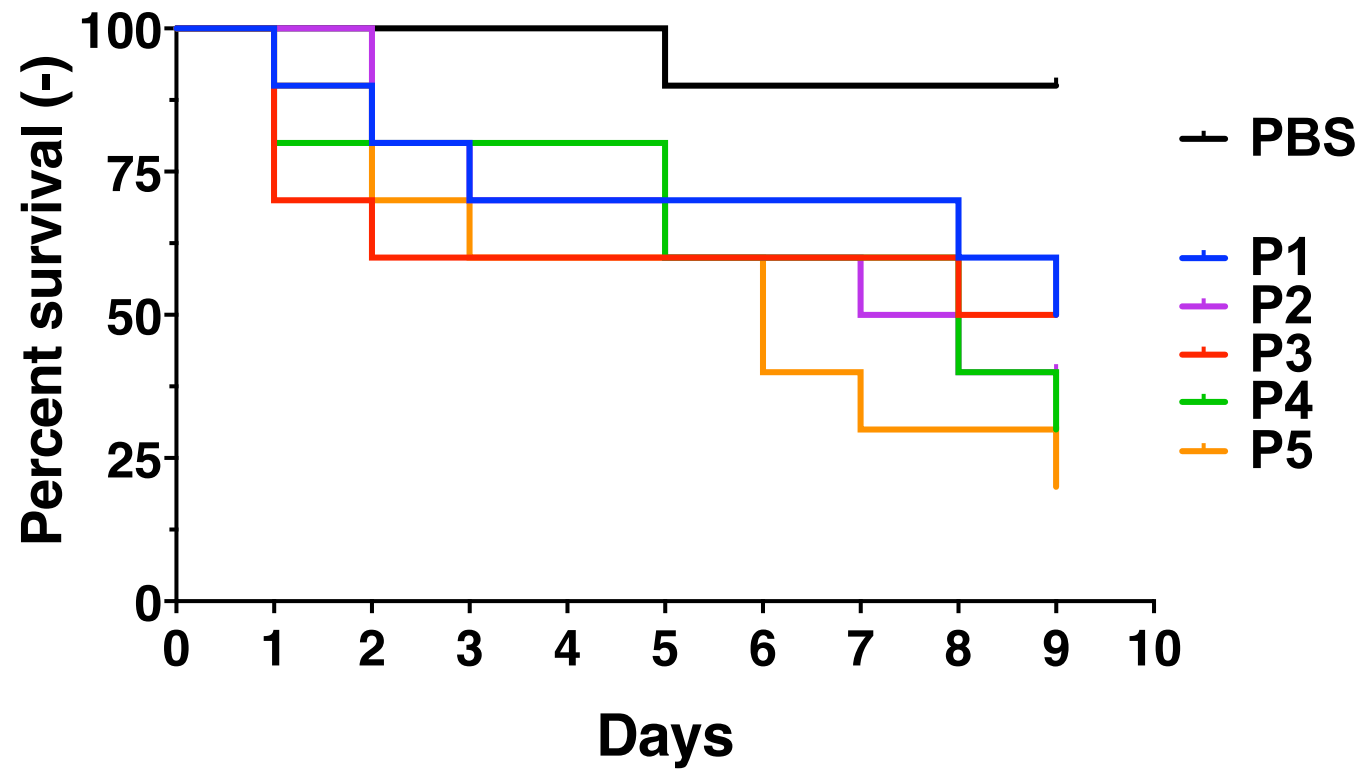

Fig S2

Supplement: FIG S2 [file mBio.02512-19-sf002.pdf]
