# Supplementary material for: Comparative Genomics for the Elucidation of Multidrug Resistance in Candida lusitaniae
Source: mBio. 2019 Dec 24;10(6):e02512-19. doi: 10.1128/mBio.02512-19 (PMC6935856; doi:10.1128/mBio.02512-19)
Supplement: TABLE S1 [file mBio.02512-19-st001.docx]

**Table S1 : Primers and guides used in this study**

| **Primers** | **Sequence** |
| --- | --- |
| ClMRR1-SacI | TAGGAAGAGCTCACAATAAGTTTTCATTG |
| ClMRR1-SacII | TTTGTCCGCGGTTCTGGTTCTATATGAG |
| ClMRR1-Apa | TTGTGGGCCCAGAGATCTGTTCTAATGA |
| ClMRR1-XHO | TCTTCTCGAGGCAATTACATGTCGTGT |
| ClMRR1-verif3 | ACTAGTAAAGCTACAGACAGATTGC |
| ClMRR1-Xhorev | AGGCTCTCGAGGAAGAGCTTACAATAA |
| MFS7-Kpn | AGATGGTACCTATGATCAGCCGAAAAACAA |
| MRR1-3_rev_new | GATGAGGATGGCGAGAAGATCAATTCCATCTCCTCTACTCTTGGAACAAAGAGAATTGTCAATCCTAACTGGTACCCACTTGTTGTTTTCAACGATGACGAGACAGCAGTATAGCGACCAGC |
| ClMRR1_F | CATTGCACAAAAGCAAAGC |
| ClMRR1_2900_F | AAATACCATCTTACATGCCGACAA |
| ClMRR1_3377_R | TGTGTTGTTCGAAAAGACAGAAAG |
| MSF7-XHO | AAACTTCTCGAGCATTGCGATTAGGTATTAG |
| MFS7-SacI | TGTGGAGCTCGGCGCCGGAATCGGCCACTA |
| MFS7-SacII | GCATCCGCGGGGTTAGTAATTGAGTTCACA |
| 5-MFS7-A | AGATGGTGCATATGATCAGCCGA |
| NAT1-BglII | GCCCAGATCTCCCTCCTTGACAGTCTTGAC |
| MRR1-5_pDS2046 | GCACAGAAAGCCAGAAAAAAACAGAAATTCCAGCGAAGCACTAAAACACGACATGTAATTGCCACGAGAAGCTCGAGGAAGTTCCTATACTT |
| MRR1-3_pDS2046 | TTGCAAATGCTTTCATGTCTTAAACATACCCTTTACCAGTAAAGTATCCTTGCCAAATTTCGTTCCATAACGCTCTAGAACTAGTGGATCT |
| NAT1-Not | GGATGGCGGCCGCAGTATCGAATCGACAGC |
| NAT1_134_R | GAGCCGTAATTTTTGCTTCG |
| Forward_gateway_MFS7 | GGGGACAAGTTTGTACAAAAAAGCAGGCTTGATGCTCAGCAAGTTTGTCAGAGAAAGTTTT |
| Reverse_gateway_MFS7 | GGGGACCACTTTGTACAAGAAAGCTGGGTCAAATCCAGAGTACTTGGATCTTGCCCTCAA |
| MFS7_XbaI | GCGCTCTAGAAAAATGCTCAGCAAGTTTGTCAGAGA |
| MFS7_Nhe | GCGCGCTAGCTTAAAATCCAGAGTACTTGGATCTT |
| ERG4-NAT1-F | CTAGCTCAGATATAGTTCTAATCCATCAAGCTTGCCTCGTCCC |
| ERG4-NAT1-R | GGGACGAGGCAAGCTTGATGGATTAGAACTATATCTGAGCTAG |
| ERG4-NAT1t-R | TAGGCAGAAATGTGAGCTAACTGGATGGCGGCGTTAGTA |
| ERG4-NAT1t-F | TACTAACGCCGCCATCCAGTTAGCTCACATTTCTGCCTA |
| ERG4-P1 | GAATGACATCTCGTATAGAATCCAGAA |
| ERG4-P2 | GCTACTGTTGAGTTTGCAATTA |
| ERG4-P3 | ATCGCGTTTAGAACTTATGTGCAC |
| ERG4-P4 | CTGCATTGAGCTCAGTGTT |
| ERG3-HYG-F | CAAGTGGGTTTCTGTTTGTCTATAGTGCTTGCTGTTCGATAT |
| ERG3-HYG-R | ATATCGAACAGCAAGCACTATAGACAAACAGAAACCCACTTG |
| ERG3-HYGt-F | ATCCCATTCATTCCATCATAAAATCAAGCGCAGATTGTCTATTC |
| ERG3-HYGt-R | GAATAGACAATCTGCGCTTGATTTTATGATGGAATGAATGGGAT |
| ERG3-P1 | CACCAGTGCGATGTATACGACTT |
| ERG3-P2 | AGTCTACTTTCGTAAACTCGTTTTA |
| ERG3-P3 | GCATAATACTAACAAACCTCAGGAC |
| ERG3-P4 | ATACAAGACGCCTCACAAAAAT |
| ClERG4_1427_F | CGGATCTCGTCAACCCTTCT |
| ClERG3_1,104_F | CTTGTTGTTGTTCACCTTTGTCAA |
|  |  |
| **Guides** | **Sequence** |
| crMRR1 | AATCAAGAGAATGCCCACGG |
| crMFS7 | TCCATTATTCGGTTCAATCT |
| crRNA_MRR1_rev5 | ACTAACAGTGAACATGCTTG |
| crRNA_MRR1_rev3 | TTTTCAACGATGACGAGTGA |
| crMRR1_del5 | AGATTCCGCCTCTATCGCAG |
| crMRR1_del3 | TGTATCGAAATGGTTTACGA |
| CrERG4 | GGTTTGCATCGCCTTTCCCT |
| CrERG3 | ACGGTCAATTTACCACCTTG |

**Table S2: Strains used in this study**

| Strain name (alias) | Parental strain | Genotype | Reference |
| --- | --- | --- | --- |
| SC5314 | - | wild type |  |
| DSY4684 | STY31 | *cdr2*A∆::FRT/*cdr2*B∆::FRT *cdr1*A∆::FRT/*cdr1*B∆::FRT *ura3*∆::FRT/*ura3*∆::FRT *mdr1*∆ ::hisG/*mdr1*∆::hisG | (1) |
| DSY5170 | DSY4684 | *cdr2*A∆::FRT/*cdr2*B∆::FRT *cdr1*A∆::FRT/*cdr1*B∆::FRT::pDS2022 *ura3*∆::FRT/*ura3*∆::FRT *mdr1*∆ ::hisG/*mdr1*∆::hisG | This study |
| ANY-MDR1-GFP | DSY4684 | *cdr2*A∆::FRT/*cdr2*B∆::FRT *cdr1*A∆::FRT/*cdr1*B∆::FRT::pDS1874 *ura3*∆::FRT/*ura3*∆::FRT *mdr1*∆ ::hisG/*mdr1*∆::hisG | (1) |
| DSY5169 | DSY4684 | *cdr2*A∆::FRT/*cdr2*B∆::FRT *cdr1*A∆::FRT/*cdr1*B∆::FRT *ura3*∆::FRT/*ura3*∆::FRT *mdr1*∆ ::hisG/*mdr1*∆::hisG RP10::Clp10 |  |
| CPY41 | SC5314 | *ura3*Δ/*ura3*Δ *ADH1/adh1*::pNIMX | This study |
| DSY5194 | CPY41 | *ura3*Δ/*ura3*Δ *ADH1/adh1*::pNIMX RP10::pDS2034 | This study |
| DSY4606 (P1) ^a^ | - | wild type | (2) |
| DSY4590 (P2) ^a^ | DSY4606 (P1) |  | (2) |
| DSY4661 (P3) ^a^ | DSY4606 (P1) |  | (2) |
| DSY4593 (P4) ^a^ | DSY4606 (P1) |  | (2) |
| DSY4662 (P5) ^a^ | DSY4606 (P1) |  | (2) |
| DSY5240 | DSY4593 (P3) | *mrr1*∆::*NAT1* | This study |
| DSY5242 | DSY4606 (P1) | *mrr1*∆::*NAT1* | This study |
| DSY5246 | DSY4593 (P3) | *mfs7*∆::*NAT1* | This study |
| DSY5248 | DSY4606 (P1) | *mfs7*∆::*NAT1* | This study |
| DSY5405 | DSY4593 (P3) | *mrr1*∆::*NAT1*-FLIP ^b^ | This study |
| DSY5416 | DSY5405 | *mrr1*∆::FRT | This study |
| DSY5437 | DSY5416 | *mrr1*∆::FRT::*MRR1*::*NAT1* | This study |
| DSY5438 | DSY5416 | *mrr1*∆::FRT::*MRR1*^V668G^::*NAT1* | This study |
| DSY5441 | DSY4590 | *erg4^amber^*::*ERG4*::*NAT1* ^c^ | This study |
| DSY5444 | DSY4662 | *erg4 ^amber^*::*ERG4*::*NAT1 erg3 ^ochre^* ^c^ | This study |
| DSY5452 | DSY5444 | *erg4 ^amber^*::*ERG4*::*NAT1* *erg3 ^ochre^*::*ERG3*::*CaHygB* ^d^ | This study |

^a^: Isolates are given the strain collection number DSY and a short designation (P1-P5) used earlier (2).

^b^: FLIP denotes the cassette FRT-p_MAL2_-CaFLP-*NAT1*-FRT.

^c^: *erg4^amber^* denotes a change of the TCG codon (Ser^412^) into a stop codon (TAG).

^d^: *erg3^ochre^* denotes a change of the CAA codon (Gln^308^) into a stop codon (TAA).

**References**

1. Banerjee A, Khandelwal NK, Sanglard D, Prasad R. 2016. A New Endogenous Overexpression System of Multidrug Transporters of Candida albicans Suitable for Structural and Functional Studies. Front Microbiol 7:261.

2. Asner SA, Giulieri S, Diezi M, Marchetti O, Sanglard D. 2015. Acquired Multidrug Antifungal Resistance in Candida lusitaniae during Therapy. Antimicrob Agents Chemother 59:7715-22.
